# The Mechanisms of lncRNA-Mediated Multidrug Resistance and the Clinical Application Prospects of lncRNAs in Breast Cancer

**DOI:** 10.3390/cancers14092101

**Published:** 2022-04-23

**Authors:** Pingting Ye, Lei Feng, Shuo Shi, Chunyan Dong

**Affiliations:** Department of Oncology, Shanghai East Hospital, School of Medicine, Shanghai Key Laboratory of Chemical Assessment and Sustainability, School of Chemical Science and Engineering, Tongji University, Shanghai 200120, China; yepingting1996@163.com (P.Y.); 2005fenglei_101@163.com (L.F.)

**Keywords:** MDR, lncRNA, breast cancer, chemoresistance, chemotherapy, exosome

## Abstract

**Simple Summary:**

Multidrug resistance (MDR) is a major cause of breast cancer (BC) chemotherapy failure. Long noncoding RNAs (lncRNAs) have been shown closely related to the chemoresistance of BC. In this work, the mechanisms of lncRNA-mediated MDR in BC were elaborated from eight sections, including apoptosis, autophagy, DNA repair, cell cycle, drug efflux, epithelial-mesenchymal transition, epigenetic modification and the tumor microenvironment. Additionally, we also discuss the clinical significance of lncRNAs, which may be biomarkers for diagnosis, therapy and prognosis.

**Abstract:**

Breast cancer (BC) is a highly heterogeneous disease and presents a great threat to female health worldwide. Chemotherapy is one of the predominant strategies for the treatment of BC; however, multidrug resistance (MDR) has seriously affected or hindered the effect of chemotherapy. Recently, a growing number of studies have indicated that lncRNAs play vital and varied roles in BC chemoresistance, including apoptosis, autophagy, DNA repair, cell cycle, drug efflux, epithelial-mesenchymal transition (EMT), epigenetic modification and the tumor microenvironment (TME). Although thousands of lncRNAs have been implicated in the chemoresistance of BC, a systematic review of their regulatory mechanisms remains to be performed. In this review, we systematically summarized the mechanisms of MDR and the functions of lncRNAs mediated in the chemoresistance of BC from the latest literature. These findings significantly enhance the current understanding of lncRNAs and suggest that they may be promising prognostic biomarkers for BC patients receiving chemotherapy, as well as therapeutic targets to prevent or reverse chemoresistance.

## 1. Introduction

Breast cancer (BC), as one of the most common malignant tumors, affects 30% of adult women worldwide [1]. For the treatment of BC, chemotherapy is one of the predominant strategies and has remarkably decreased mortality. However, multidrug resistance (MDR) is a leading cause of BC chemotherapy failure [2], and the complex mechanisms have not been fully elucidated. Long noncoding RNAs (lncRNAs) have been demonstrated to play prominent roles in many critical cellular processes, such as transcription, translation, epigenetic control, stem cell differentiation, cell autophagy and apoptosis [3]. Therefore, exploring the mechanisms of lncRNA-mediated MDR in BC has become a hot research topic in the last few years [4,5].

**Figure 1:**
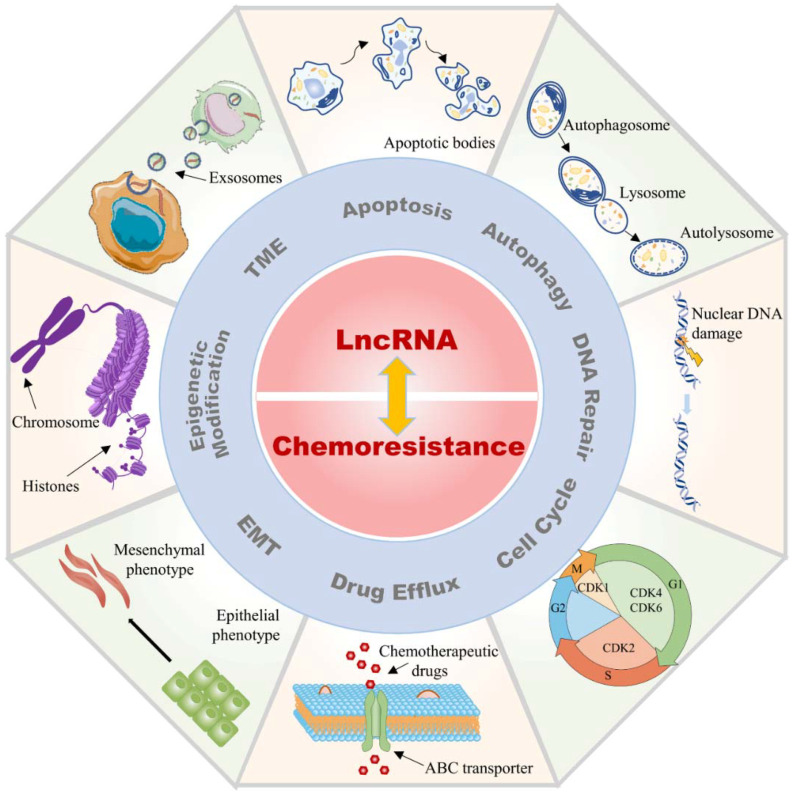
Overview of the relationship between lncRNAs and chemoresistance in this review.

In this review, we summarize the latest research exploring the mechanisms of MDR and the functions of lncRNAs in BC and then elaborate on the relationship between them in eight sections (Figure 1), including apoptosis, autophagy, DNA repair, cell cycle, drug efflux, epithelial-mesenchymal transition (EMT), epigenetic modification and the tumor microenvironment (TME). We also discuss the potential applications of lncRNAs as biomarkers for diagnosis, therapy and prognosis, which may lay a foundation for the clinical development of improved strategies to overcome the chemoresistance of BC. Finally, we present a brief future outlook on the challenges and opportunities for lncRNAs in BC therapy.

## 2. MDR

MDR is a phenomenon in which cancer cells exhibit reduced sensitivity to several kinds of drugs with different mechanisms [6]. According to the responsiveness of drugs, chemoresistance can be classified as intrinsic (i.e., existing before chemotherapy) and acquired resistance (i.e., developing during chemotherapy) [2,7]. Additionally, most chemotherapeutic drugs (including classical cytotoxic drugs and molecular targeted drugs) have a narrow therapeutic range. To promote clinical outcomes, it is necessary to identify the underlying molecular mechanism of MDR and bypass this barrier. To date, a large number of attempts have been made to overcome MDR, such as MDR transporter inhibitors [8], nanomedicines [9], small interfering RNAs (siRNAs) [10], and monoclonal antibodies [11]. Although some of them have already entered clinical trials, the effects are still unsatisfactory [12]. Many critical issues remain, including low therapeutic responses, drug-drug interactions, clinical trial design problems and high cytotoxicity [8,13,14,15]. Therefore, more new and effective therapeutic strategies are urgently needed.

## 3. LncRNAs

**Figure 2:**
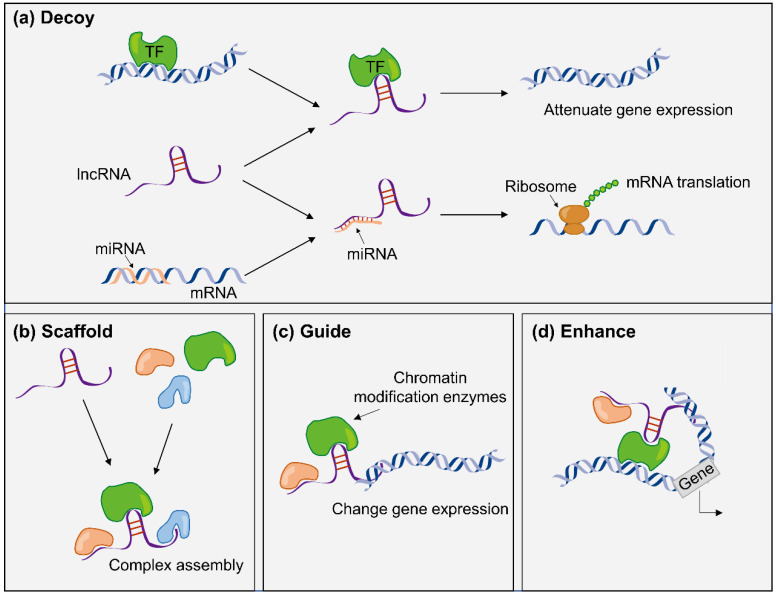
Models of lncRNA mechanisms of action. (**a**) lncRNAs may act as decoys to lead transcription factors (TFs) away from DNA targets or directly bind to sequester complementary RNA transcripts, such as miRNAs (also known as competing endogenous RNAs or “sponges” of miRNAs). The effect of this biological function is to regulate the expression of the genes and the translation of the mRNA. (**b**) lncRNAs may act as scaffolds to assemble two or more proteins into a complex. (**c**) lncRNAs may act as guides to regulate gene expression by recruiting proteins, such as chromatin modification enzymes. (**d**) lncRNAs may act as enhancers in chromosome looping (also known as cis-regulatory elements) [23].

As a subclass of noncoding RNAs, lncRNAs consist of more than 200 nucleotides and are usually divided into 5 categories according to their location relative to adjacent protein-coding genes, including bidirectional, antisense, intergenic, intronic, and sense lncRNAs [16]. Although lncRNAs do not encode functional proteins, they exert biological functions by serving as to regulate the expression of multiple target genes in cellular processes and diseases [17,18,19] (Figure 2 shows the biological functions of lncRNAs in gene regulation). With the development of detection technologies, circulating lncRNAs in bodily fluids may serve as valuable diagnostic and prognostic markers. For example, the lncRNA PCA3 in urine has been approved by the US Food and Drug Administration (FDA) as a urine marker for prostate cancer because of its better sensitivity and specificity than prostate-specific antigen (PSA) [20,21,22]. The following main advantages and drawbacks of lncRNAs serving as biomarkers have been objectively identified.

### 3.1. The Advantages of lncRNAs

(1) Some circulating lncRNAs are relatively stable in bodily fluids [24,25]. (2) In addition to tumor tissues, lncRNAs can also be detected in different types of body fluids (e.g., blood, urine [20], saliva [26] and plasma [24]). (3) Compared to tissue biopsy, the detection of lncRNAs is noninvasive and has acceptable specificity and sensitivity [25,27]. (4) Similar to most traditional biomarkers, the levels of lncRNAs show dynamic changes with the response to tumor progression [28].

### 3.2. The Drawbacks of lncRNAs

(1) Most lncRNAs have lower stability, owing to a high concentration of RNases in blood circulation [29,30]. Though fragmented lncRNAs in plasma are easily missed by standard RNA-seq, new RNA-seq will be improved for identifying more lncRNAs [31]. (2) lncRNAs are characterized as tissue-specific expression and low expression level [32]. However, new methods are investigated for improving the resolution and sensitivity [33]. (3) In previous studies, many lncRNAs are validated in vitro and vivo experiments only, but not yet in clinical population. The actual clinical value of most lncRNAs remains to be determined [25].

During the last decade, the relationship between lncRNAs and MDR has attracted extensive attention in biological research. Although some of the drawbacks are pointed out above, more methods can be used to remedy or improve them. Consequently, deciphering the mechanisms of chemoresistance by analysing the functions of lncRNAs is expected. In future clinical practice, lncRNAs may not only serve as prognostic biomarkers for MDR but also be valid targets to reverse MDR and guide clinical medication.

## 4. Transcription Factors Involving in lncRNA Regulation

As shown in Figure 2, the main function of lncRNAs in gene regulation is mediating in transcription. In this biological process, TFs are indispensable, which can activate or repress transcription by binding sequence-specific DNA. The interaction between lncRNAs and TFs can be direct or indirect by inhibition, activation, recruitment or decoy mechanism [34]. For example, lncRNA *TROJAN* was discovered in ER+ BC, relating to resistance of Cyclin-Dependent Kinase 4 and 6 (CDK4/6) inhibitor [5]. Mechanistically, the transcription of CDK2 could be regulated, owing to that *TROJAN* hindered the combination of NF-κB repressing factor (NKRF) and RELA (a TF of the NF-κB pathway). Similarly, lncRNAs, as transcription products, require the participation of TFs [35]. TFs and lncRNAs have complex network relationship; however, only a few articles have reported a small part of it. 

## 5. The Relationship between MDR and lncRNAs

### 5.1. Regulation of Cell Survival and Death

#### 5.1.1. Suppressing Apoptosis

**Figure 3:**
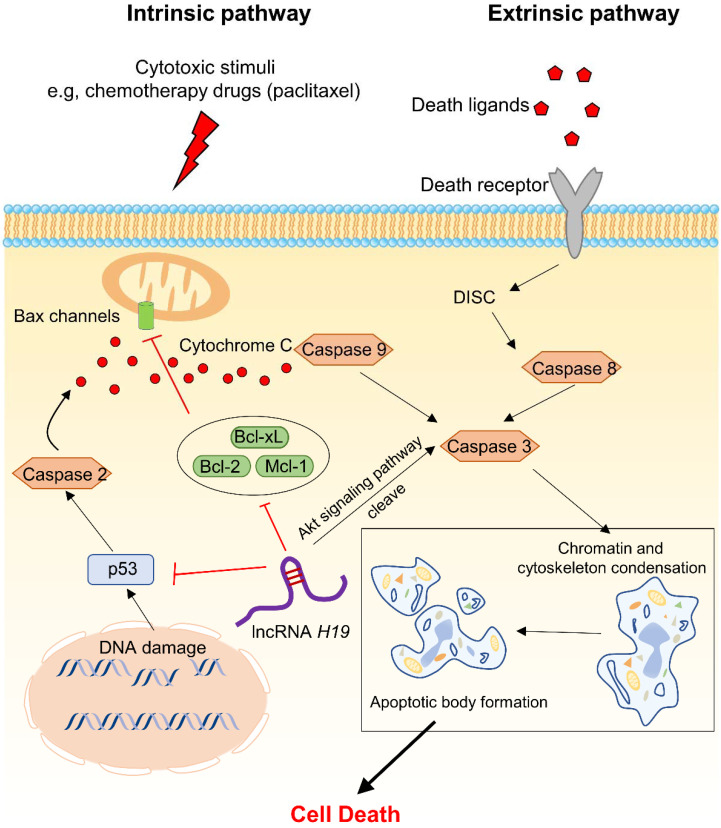
Overview of the apoptosis pathways (lncRNA *H19* is used as example clarifying the mechanism). The intrinsic pathway of apoptosis is initiated by the cell itself in response to cytotoxic stimuli. The extrinsic pathway is initiated via death receptors stimulated by death ligands. When caspase 3 is activated, the two pathways merge and lead to cell death [37]. Bax channels, Bcl-2-associated protein X channels; Bcl-2, B-cell lymphoma 2; Bcl-xL, B-cell lymphoma extra-large; Mcl-1 induced myeloid leukemia cell differentiation protein 1; DISC, death inducing signalling complex.

Apoptosis, known as programmed cell death, is the most common activation pathway in the response to chemotherapy [36]. Generally, apoptosis is mainly triggered in caspase-mediated extrinsic or intrinsic pathways (a more detailed classification is shown in Figure 3) [37,38]. Currently, most anticancer drugs used in clinical oncology induce cancer cell death through apoptotic pathways, including the p53 pathway, the Akt signalling pathway and apoptotic regulatory proteins (such as B-cell lymphoma 2 protein (Bcl-2) and Bcl-2–associated X protein (Bax)) [39,40]. Thus, activation or inactivation of apoptotic factors may lead to MDR during treatment. Many studies have shown that lncRNAs associated with apoptotic pathways are involved in MDR in several human cancers, such as glioma [41], osteosarcoma [42], ovarian cancer [43] and lung cancer [44]. For BC, as shown in Table 1, lncRNAs can also regulate apoptosis through a variety of cancer-related signalling pathways in the chemoresistance process. It has been revealed that the lncRNA *H19* is overexpressed in approximately 70% of BC patients, and it has been indicated to be an oncogenic lncRNA [45]. By comparing tissues from 60 patients, Li et al. showed that *H19* was significantly upregulated in BC tissues, especially in triple-negative breast cancer (TNBC) [46]. In their study, they concluded that p53 could be inhibited and that the expression of TNFAIP8 was increased at high levels of *H19*. This finding suggests that *H19* can regulate cellular physiological processes by modifying the p53 pathway. In addition, Han et al. compared the expression level of *H19* in paclitaxel-resistant TNBC and paclitaxel-sensitive TNBC and found that *H19* was upregulated in the former. They further confirmed that *H19* notably increased the phosphorylation of Akt and mediated the expression of key proteins in the Akt signalling pathway, including p-Akt (Ser473), Akt, Bax, Bcl-2 and cleaved caspase-3 [47]. Coincidentally, upregulation of *H19* was identified in paclitaxel-resistant estrogen receptor α (ERα)-positive BC. Si et al. reported that *H19* in ERα-positive BC attenuated cell apoptosis by downregulating the transcription of BIK and NOXA, which are members of the Bcl-2 family. Interestingly, they also revealed that *H19* was the downstream target molecule of ERα and demonstrated that ERα could regulate the progression of BC [48]. This result suggested that the clinical efficacy of paclitaxel might be influenced in ERα-positive BC due to the high expression of *H19*.

As discussed above, *H19* is involved in different apoptosis pathways, including inhibiting p53, downregulating proapoptotic proteins (e.g., BIK), upregulating antiapoptotic proteins (e.g., Bcl-2) and phosphorylating Akt. As shown in the schematic of apoptosis (Figure 3), *H19* can accelerate apoptosis processes by mediating these pathways through the opening of Bax channels and cytochrome C release. Though in different subtypes of BC, *H19* involves in similar apoptosis processes. In addition to *H19*, other lncRNAs shown in Table 1 also play similar roles and will not be discussed further here. In summary, lncRNAs mediate BC chemotherapeutic resistance by affecting apoptotic processes and can serve as potential prognostic markers and therapeutic targets for preventing apoptosis.

#### 5.1.2. Autophagy

**Figure 4:**
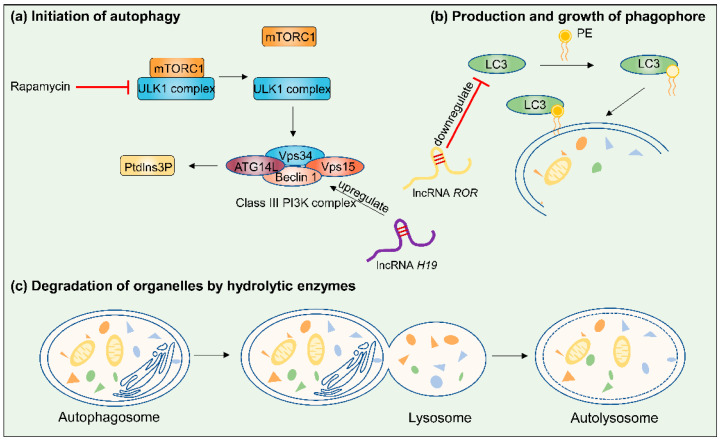
Summary of the steps involved in autophagy (lncRNA *H19* and *ROR* are used as examples for clarifying the mechanism). Autophagy is initiated by the stepwise engulfment of cellular materials by the phagophore, which sequesters materials in double-membraned vesicles known as autophagosomes [75]: (**a**) When mammalian target of rapamycin (mTOR) is inhibited, mTOR complex 1 (mTORC1) isolates from the ULK1 complex. The first step of vesicle nucleation is activating Vps34, a class III phosphatidylinositol 3-kinase (PI3K), to produce phosphatidylinositol-3-phosphate (PtdIns3P). (**b**) A part of the vesicle elongation process is to bind phosphatidylethanolamine (PE) to LC3. (**c**) The formation of autophagosomes is completed after closure of the phagophore double membrane, and then autophagosomes fuse with lysosomes, resulting in degradation of the contents.

Autophagy is a homeostatic process in which cellular materials are sequestered into autophagosomes and degraded by lysosomes [72,73,74]. Generally, autophagy is a multistep process, and the simplified steps are as follows (the specific steps are shown in Figure 4): autophagy initiation, autophagosome formation, autolysosomal fusion, and autolysosomal degradation [36,75]. In the context of tumor treatment, autophagy is widely regarded as a double-edged sword that can either facilitate or inhibit tumor progression [76]. As shown in Table 1, lncRNAs might interact with key targets to influence the efficacy of chemotherapy in the process of autophagy. Li et al. designed an original study to explore the functions of lncRNA regulator of reprogramming (*ROR*) in modulating autophagy. They found that the small interference-mediated lncRNA *ROR* could facilitate autophagy and increase sensitivity to tamoxifen by upregulating the expression of two autophagic genes—Beclin-1 and light chain 3 (LC3) [59]. In contrast, another study showed that overexpression of *H19* promoted autophagy and induced tamoxifen resistance in ERα-positive BC. Using the autophagy inhibitors 3-methyladenine and chloroquine could downregulate the expression of Beclin-1 and restore sensitivity to tamoxifen [60]. For the same drug—tamoxifen, however, the situation is quite the opposite. The key reason for this phenomenon is different types of BC cells. Beclin-1, a major mediator of autophagy, can inhibit estrogenic signalling and induce tamoxifen resistance in ERα-positive BC [77]. As we discussed in the “Suppressing apoptosis” section, *H19* can inhibit apoptosis by downregulating proapoptotic proteins and promote paclitaxel resistance in ERα-positive BC [48]. The same lncRNA acts via different mechanisms in the same type of BC. In short, the mechanisms of MDR are multifactorial and complex. There is no doubt that different lncRNAs mediate autophagy by modulating the expression of key autophagy-related genes and yield dramatically different effects in chemoresistant BC. However, the interactions of lncRNAs, autophagy processes and MDR remain unknown and need more research to clarify.

#### 5.1.3. Activating DNA Repair

Considerable evidence supports that many chemotherapeutic agents exert anticancer effects by destroying the stability of genes and activating downstream DNA damage signaling pathways [78,79]. Tumor cells may activate DNA damage repair pathways to resist DNA damage and contribute to MDR [80]. Accumulating studies have reported that lncRNAs in different human cancers are related to DNA repair in MDR [81,82,83]. It is widely accepted that phosphatase and tensin homolog (PTEN) controls DNA repair [84,85] and exerts multiple nuclear functions [86,87]. Moreover, it also participates in the key processes of genetic transmission to promote the fidelity of DNA replication [88,89,90] and chromosome segregation [91,92,93]. Jiang et al. and Li et al. reported that lncRNA growth arrest-specific transcript 5 (*GAS5*) functions as a tumor suppressor in chemoresistant BC. *GAS5* induced resistance to chemotherapeutic drugs by suppressing PTEN in two situations (different molecular subtypes of BCs and different drugs) [64,67]. In addition to PTEN, poly (ADP-ribose) polymerase (PARP) also participates in the DNA repair process, serving as an enzyme to repair single-stranded breaks [94]. Wang et al. reported that *H19* plays a crucial role in doxorubicin-resistant BC by downregulating PARP1 [69]. In the clinic, resistance to PARP inhibitors is common. Ideally, knockdown of *H19* might increase the sensitivity of BC cells to doxorubicin and PARP inhibitors. This implies that targeting lncRNAs could reverse resistance, increase the effectiveness of treatment strategies, and achieve good clinical efficacy. As shown in Table 1, although many lncRNAs participate in DNA repair via distinct pathways in the chemoresistance of BC, they are the tip of the iceberg. It is impossible to achieve clinical translation based on the currently available information. There is still a long way to go to fully clarify the relationship between lncRNAs and DNA repair.

### 5.2. Regulating the Cell Cycle

**Figure 5:**
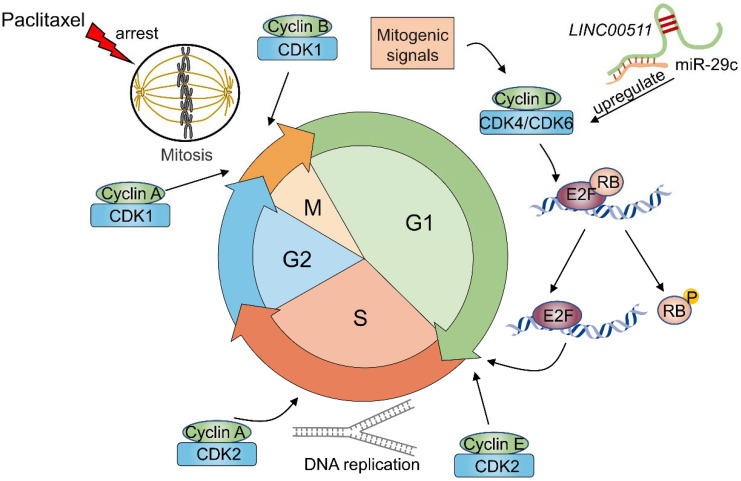
Cell cycle progression and CDKs (*LINC00511* is used as example for clarifying the mechanism). The cell cycle is divided into four distinct phases: G1 (postmitotic interphase), S phase (DNA synthesis phase), G2 (postsynthetic phase), and M phase (mitosis). Mitogenic signals activate CDK4 and CDK6 complexes to initiate the phosphorylation (P) of key substrates, including the tumor suppressor retinoblastoma protein (RB), thereby releasing a gene expression program that is coordinated by the E2F family of transcription factors. The subsequent activation of CDK2-Cyclin A and CDK2-Cyclin E complexes initiates DNA replication. With the completion of DNA replication, CDK1–Cyclin A and CDK1–Cyclin B complexes form to phosphorylate targets in G2 phase. In the absence of DNA damage and following proper preparation for chromosomal segregation, the cellular default is to activate CDK1–Cyclin B complexes and progress into mitosis [98]. CDK, cyclin-dependent Ser/Thr kinase.

Faithful transmission of genetic information requires not only timely ordered execution and integration of DNA replication but also accurately controlled cell cycle transitions [95]. To complete the essential task of genetic transmission, cells must precisely complete the typical cell cycle, which consists of four distinct phases known as the G1, S, G2, and M phases [96]. Commonly, cancer is characterized by aberrant activity of the cell cycle resulting in uncontrolled tumor cell proliferation. As illustrated in Figure 5, the cell cycle is driven by a number of regulatory factors, including cyclins and cyclin-dependent Ser/Thr kinases (CDKs) [97,98,99]. Among them, CDKs exert critical functions via periodic activation and inactivation. To date, many chemotherapeutic drugs have anticancer effects by targeting the cell cycle. As one of the most effective and widely used anticancer drugs, paclitaxel is mainly applied in patients with ovarian cancer or BC and exerts its action mainly by disrupting normal microtubule dynamics and inducing cell cycle arrest at the G2/M phase transition [100,101,102,103]. Zhang et al. reported that *LINC00511*, working as a molecular sponge of miR-29c, induced paclitaxel resistance in BC cells [104]. In their study, they confirmed that CDK6 was upregulated as a target of miR-29c. CDK6 is known as a cyclin D1-dependent kinase that facilitates the G1/S phase transition [105,106] (Figure 5). Zhang et al. showed that downregulation of CDK6 attenuated the regulatory effect of miR-29c on paclitaxel cytotoxicity in BC cells. In another study, CDK6 was also upregulated in trastuzumab-resistant BC through a distinct pathway—the lncRNA *UCA1*/miR-18a/YAP1 pathway [65]. The upregulation of CDK6 accelerated the cell cycle, attenuated the effect of paclitaxel or trastuzumab and eventually contributed to chemoresistance. Thus, different lncRNAs may have similar mechanisms in different conditions via the same function. As shown in Table 2, lncRNAs are involved in the key regulatory factors that regulate the cell cycle of BC patients receiving chemotherapy. CDKs play a central role in controlling the cell cycle, which increases the possibility of devising therapeutic strategies based on their medicinal properties. At present, the FDA has approved CDK 4/6 inhibitors (including palbociclib, ribociclib and abemaciclib) for HR+ advanced BC [107]. Resistance to this class of drugs inevitably emerges after long-term treatment [107,108]. As a result, cell cycle-related lncRNAs may be targets for abrogating chemoresistance and enhancing the prognosis of BC patients.

### 5.3. Drug Efflux

Drug efflux is regarded as the predominant cause of MDR in human cancers. Hydrophobic chemotherapeutic drugs can be pumped out of tumor cells via the ATP-binding cassette (ABC) transporter superfamily, thereby reducing the effectiveness of the drugs and possibly resulting in tumor recurrence [149]. To date, according to their sequence homology and structural similarities, a total of 48 human ABC transporter genes have been divided into seven subfamilies (ABCA to ABCG) [150]. Among the ABC transporter superfamily, P-glycoprotein (P-gp/ABCB1), multidrug resistance protein 1 (MRP1/ABCC1), and breast cancer resistance protein (BCRP/ABCG2) are considered to be the most closely related to MDR in cancer cells [149,151]. Recently, a number of studies have shown that lncRNAs play a key role in increasing the outflow of a wide range of chemotherapeutic agents from human cancer cells, such as esophageal squamous cell carcinoma [152], osteosarcoma [153], and hepatocellular carcinoma [154]. A similar function of lncRNAs has also been explored in BC (Table 2). For instance, Chen et al. found that *GAS5* was downregulated in adriamycin-resistant BC cells, while the mRNA ABCB1 was upregulated based on the RNA expression profiles. Further investigating the related mechanism in detail, *GAS5* regulates its target Dickkopf 2 (DKK2) by working as a molecular sponge of miR-221-3p and inhibiting activation of the Wnt/β-catenin pathway [119]. The promoter of the ABCB1 gene contains TCF4/LEF binding motifs, which are targets of β-catenin/TCF4 transcriptional regulators [155]. Therefore, the downregulation of *GAS5* will disinhibit the Wnt/β-catenin pathway, increase the expression of ABCB1, and promote the exit of adriamycin from intracellular sources. With the function of regulating drug efflux metabolism, targeting lncRNAs may become a promising approach to eliminate or suppress MDR by reducing drug efflux from tumor cells. Ideally, the combination of chemotherapeutic drugs and lncRNA target drugs can reduce the dose and side effects for BC patients.

### 5.4. Modulating the EMT Process

**Figure 6:**
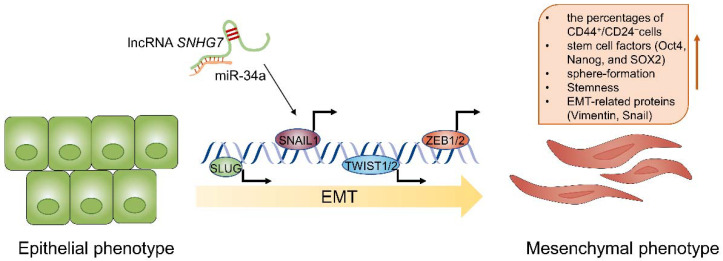
Scheme of the proposed mechanism related to lncRNA *SNHG7* in EMT process. *SNHG7*, as a molecular sponge of miR-34a, mediating EMT process, which is driven by EMT-transcription factors (SLUG, SNAIL1, TWIST1/2, ZEB1/2) that repress epithelial marker genes and activate mesenchymal marker genes. EMT, Epithelial–mesenchymal transition; *SNHG7*, small nucleolar RNA host gene 7.

EMT is a reversible dynamic process that enables various biochemical changes to occur in polarized epithelial cells and presents a mesenchymal cell phenotype, thereby improving apoptosis resistance and enhancing migration and invasion abilities [156]. Since the link between EMT and MDR was proposed in the early 1990s, the role of EMT in MDR has attracted great attention [157]. There is a growing appreciation that MDR is frequently associated with EMT in different types of cancers, including pancreatic cancer [158], bladder cancer [159] and breast cancer [160]. Several EMT-transcription factors (TFs) have been identified as master regulators of EMT, which can typically be classified into three different protein families—the ZEB (including ZEB1 and ZEB2), Snail (including Snail and Slug), and basic helix–loop–helix (including TWIST1, TWIST2, and TCF3) families [161]. These EMT-TFs are involved in many signalling pathways, such as the NF-ĸB [162], Notch [163], Wnt [164], Hedgehog [165], AKT-mTOR [166] and MAPK/ERK [167] pathways. It is commonly accepted that lncRNAs are associated with these EMT-TFs and signalling pathways. The functions of lncRNAs in the EMT process are critical to chemoresistant BC (Table 2). Sun et al. reported that the lncRNA small nucleolar RNA host gene 7 (*SNHG7*) was an oncogenic lncRNA that acts as a molecular sponge of miR-34a in BC. Furthermore, compared with the sh-NC group, they found that the EMT-related proteins vimentin and Snail were decreased in the sh-*SNHG7* group, while E-cadherin was increased [168]. This result indicated that the expression of *SNHG7* increased the expression of EMT-TFs and promoted the EMT process, eventually causing tumor progression (Figure 6).

Moreover, activation of EMT induces cancer cell resistance to multiple therapeutic drugs—another important property of cancer stem cells (CSCs). CSCs represent a subset of cancer cells with tumor formation, self-renewal, multiple differentiation, therapeutic resistance, tumor progression, relapse and metastasis [169]. Notably, activation of EMT enables non-CSCs to transform into CSCs [170]. In both preclinical and clinical samples, some studies have revealed that chemotherapy successfully eliminates the majority of non-CSCs while leaving behind a considerable number of CSCs [171,172,173]. Recently, Li et al. further explored the potential mechanism of *SNHG7* in chemoresistant BC. They found that upregulation of *SNHG7* increased the percentages of CD44^+^/CD24^−^ cells and the expression of stem cell factors (Oct4, Nanog, and SOX2) and promoted sphere-formation (Figure 6). The results of their study showed that *SNHG7* can increase stemness in chemoresistant BC via miR-34a [135].

Overall, lncRNAs are involved in EMT progression and tumor stemness and influence the sensitivity of BC cells to chemotherapeutic drugs. A full understanding the mechanisms of EMT and the functions of lncRNAs is needed for developing new strategies to prevent EMT in patients receiving chemotherapy.

### 5.5. Epigenetic Modification

**Figure 7:**
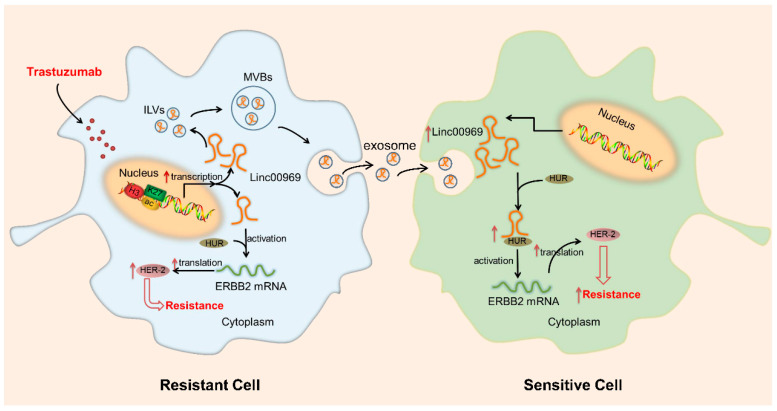
Scheme of the proposed mechanism related to *Linc00969* in trastuzumab-resistant breast cells. *Linc00969* induces trastuzumab resistance by binding to the HUR protein and promoting the translation of ERBB2 mRNA. In addition, extracellular *Linc00969* from trastuzumab-resistant cells was packaged into exosomes and disseminated trastuzumab resistance in trastuzumab-sensitive cells [137]. ILVs, intraluminal vesicles; MVBs, multivesicular bodies; HUR, Hu antigen R.

Epigenetic factors, such as chromatin remodeling and DNA methylation, are related to the spatial and temporal regulation of gene expression [174,175]. Therefore, a malignant phenotype may be induced by aberrant expression patterns or genomic alterations in chromatin remodelers. Although it has been reported that epigenetic factors contribute greatly to drug tolerance [176,177,178], most of the exact mechanisms behind these associations remain elusive. In this section, we summarized that lncRNAs regulate gene expression via epigenetic modification in chemoresistant BC cells (Table 2). As shown in Figure 7, the expression of *Linc00969* was upregulated in trastuzumab-resistant cells [137]. Then, *Linc00969* could increase the translation of ERBB2 mRNA by binding to the Hu antigen R (HUR) protein. Therefore, the protein level of HER-2 was upregulated and, subsequently, induced trastuzumab resistance in HER-2+ BC cells. As one of the most common epigenetic modifications, histone acetylation can neutralize lysine’s positive charge to relax the chromatin structure and enhance transcriptional activity [179]. It has been reported that *ZNF649-AS1*, upregulated by H3K27ac modification, confers trastuzumab resistance by binding PTBP1 and upregulating ATG5 transcription [61]. Similarly, Dong et al. used chromatin immunoprecipitation (ChIP) assays and found that lncRNA *SNHG14* can modulate H3K27 acetylation at the promoter region of the PABPC1 gene and can increase the transcription of PABPC1 [142]. Increasing the expression of PABPC1 activates the Nrf2 pathway and, then, promotes tumorigenesis and trastuzumab resistance in BC cells. Consequently, even in the same cell line exposed to the same treatment, different lncRNAs may have similar functions (e.g., guides) via different pathways. In brief, lncRNAs could play a critical biological function in regulating the expression of genes. Further research is needed to explore the deeper underlying mechanism of epigenetic modification-related lncRNAs in MDR.

### 5.6. Modifying the TME via Exosomal lncRNAs

The TME is a complex system comprising tumor cells, stromal cells (cancer-associated fibroblasts, endothelial cells, and macrophages), extracellular matrix, and soluble factors (hormones, cytokines, and enzymes) [180]. The TME not only plays an important role in the process of tumorigenesis, proliferation, and metastasis but also has a profound impact on chemotherapeutic efficacy. Exosomes, ranging in size from 20 to 150 nm, are membrane-derived vesicles originating from endosomal multivesicular bodies (MVBs) and play an essential role in TME. They can transfer useful information from host cells to recipient cells, such as lipids, proteins, microRNAs (miRNAs), messenger RNAs (mRNAs), and lncRNAs [181,182,183]. Thus, exosomal lncRNAs have been investigated to explore the mechanisms of MDR in different types of tumors, such as renal cancer [184], lung cancer [185], esophageal squamous cell carcinoma [186], BC (Table 3), gastric cancer [187], ovarian cancer [188], and cervical cancer [189].

Until now, few studies have addressed the link between exosomal lncRNAs and chemoresistance in BC (Table 3). As shown in Figure 7, Liu et al. reported that exosomes from trastuzumab-resistant cells packaged extracellular *Linc00969* and transferred it to trastuzumab-sensitive cells, which also resulted in upregulation of the HER-2 protein and induced resistance of recipient cells [137]. It has been reported that exosomes produced by tamoxifen-resistant LCC2 cells containing more *UCA1* are incorporated into MCF-7 cells and then significantly increase tamoxifen resistance in ERα-positive BC cells [190]. Notably, lncRNAs in exosomes derived from chemoresistant BC cells could confer resistance to sensitive cells, even in different cell lines. Additionally, the infiltration of immune cells into TME plays an indispensable role in the anti-tumor process. Ni et al. reported that the expression of CD73 on γδT cells (a predominant type of regulatory T cells) could be upregulated by lncRNA *SNHG16*, which is transmitted via BC-derived exosomes [191]. This is closely related to unfavorable pathological characteristics and a poor prognosis of BC. Based on these reports, transmission of exosomes might provide a new idea for drug therapy, which could change the susceptibility of cells to chemotherapeutic drugs or reverse the immunosuppressive microenvironment for more effective immunotherapy.

**Table 3:** The role of exosomal lncRNAs in drug resistance in breast cancers.

LncRNA	Type	Genomic Location	Expression Level *	Resistant Drugs	Cell Lines	Possible Mechanism ^§^	Reference
*LINC00969*	Oncogene	N/A	↑	trastuzumab	SKBR-3; BT474	↑ translation and stability of ERBB2 mRNA	[137]
*H19*	Oncogene	chr11p15.5	↑	doxorubicin	MCF-7; MDA-MB-231	N/A °	[192]
*HISLA*	Oncogene	chr14q31.3	↑	docetaxel	MDA-MB-231; BT-474; MDA-MB-468; MCF-7	inhibit the hydroxylation and degradation of HIF-1α	[193]
*AGAP2-AS1*	Oncogene	chr12q14.1	↑	trastuzumab	SKBR-3; BT474	N/A	[194]
*UCA1*	Oncogene	chr19q13.12	↑	tamoxifen	MCF-7; LCC2	↓ cleaved caspase-3	[190]

* The expression in resistant BC lines is indicated by arrows: ↑ for higher expression and ↓ for lower expression. **^§^** The effect of lncRNAs on associated pathways, miRNAs, genes, or transcription factors involved in resistance mechanisms are indicated by arrows: ↑ induction and ↓ repression. ° N/A, information not available.

Additionally, the utility of exosomes as minimally invasive liquid biopsies is particularly promising because of their presence in all biological fluids and their potential for multicomponent analyses [195]. Therefore, exosomal lncRNAs can serve as early diagnostic biomarkers and potential molecular targets for patients with MDR. However, it is worth noting that the period for the study of chemoresistance-related exosomal lncRNAs is short, and there are still many questions to be answered in the future.

## 6. The Relationship between lncRNAs and Immunotherapy

In recent years, immunotherapy is emerging as an attractive option for cancer patients, which can be divided into two types: passive and active. The outcomes of the dynamic interplay between the host immune system and tumor cells determine the effectiveness of immunotherapy. Gradually, it has been revealed that lncRNAs may serve as key regulators in this dynamic interplay, which is known as immune-related lncRNAs (IRLs) affecting immune response and disease progression.

For the host immune system, IRLs involve in differentiation and activation of immune cells, such as macrophages [196], T cells [191] and NK cells [197]. Huang et al. reported that the upregulation of NF-κ B-interacting long noncoding RNA (*NKILA*) inhibits NF-κ B activity, inducing tumor-specific cytotoxic T lymphocytes (CTLs) and type 1 helper T (TH1) cells to be more sensitive to activation-induced cell death (AICD) [198]. It suggested that regulating the expression of IRLs of immune cells might change their outcomes, which might be a novel way for transferring tumor immune microenvironment or immunotherapy resistance.

For tumor cells, IRLs involve in tumor immune evasion via regulating antigenicity [199] or the expression of immunoregulating molecules [200]. In TNBC, the expression of long intergenic noncoding RNA for kinase activation (*LINK-A*) mediates phosphorylation of TRIM71, contributing to degrade peptide-loading complex (PLC) components, Rb and p53 [199]. Consequently, the antigenicity of tumor and the capability of immunosurveillance are downregulated, contributing to affect the effectiveness of immunotherapy. It suggested that regulating IRLs of tumor might be another new way to decrease tumor immune evasion and resistance of immunotherapy.

Through biological database analysis, lots of IRLs were found to be associated with the infiltration of immune cells in BC [201,202]. Though the mechanisms correlated with most of them have not been further studied, they can also be considered as signatures to predict prognosis of BC. Based on these reports, it is concluded that IRLs can be biomarkers for predicting immunotherapy response and be targets for overcoming immunotherapy resistance.

## 7. The Prospective Clinical Application of lncRNAs for Overcoming MDR in BC Patients

### 7.1. Association of lncRNAs and Patients with BC

Indeed, existing studies are not limited to in vitro cellular and animal experiments. Many studies have also explored the relationship between lncRNAs and patients [119,137,192]. The following two situations are common: In the first case, significant differences were found in BC tissues from patients, and further experimental verification was carried out. For instance, Chen et al. collected 26 BC tissue samples from patients and compared the expression of *GAS5* and *ABCB1* between tissues from responders and nonresponders [119]. Then, they verified that *GAS5* and *ABCB1* expression was downregulated in chemoresistant patients and cell lines, indicating a positive correlation. In the second case, significant differences were first found by in vitro cellular and animal experiments and then further validated in BC patients. For instance, lncRNA *H19* was upregulated in doxorubicin-resistant cells, as reported by Wang et al. [192]. Then, they verified this result in BC patients by statistical analysis and reported that the exosomal lncRNA *H19* may be a noninvasive biomarker for doxorubicin-resistant BC patients. Briefly, some lncRNAs that are differentially expressed in cell lines are also consistent in the BC tissues of patients who received chemotherapy. The limitations of these studies are related to the small samples of patients and the lack of statistical analysis of sensitivity and specificity for lncRNAs serving as biomarkers. To achieve clinical application, further experiments and larger-scale clinical trials are needed.

### 7.2. The Potential Roles of lncRNAs in Clinical Applications

To improve cure rates and reduce mortality, early detection, early diagnosis and early treatment are recommended for BC patients. With the development of imaging techniques, various screening tools have been applied to detect and diagnose BC, such as mammography, magnetic resonance imaging (MRI), positron emission tomography (PET), computed tomography (CT), and single-photon emission computed tomography (SPECT) [203]. However, the sensitivity and specificity of these imaging techniques remain challenges for clinical application. As discussed above, chemoresistance-related lncRNAs might be potential biomarkers to predict chemotherapeutic response in BC. As previously reported by Si et al., oncogenic H19 could be regulated by ERα and induce paclitaxel resistance in ERα-positive BC [48]. In turn, the expression level of *H19* might be used to determine whether chemoresistance occurs and the degree of resistance.

Advanced detection technologies have been applied to quantify lncRNAs, including microarrays, RNA-seq, and qRT–PCR [27]. To achieve clinical application, however, there are still several limitations that need to be overcome. From a technical point of view, the procedures of sample preparation, lncRNA extraction and detection should be standardized [204,205]. In addition, the accuracy and stability of the detection results should be ensured. From a clinical standpoint, it is most important to ensure the sensitivity and specificity of lncRNAs. In addition, not only material and instrument costs but also time and labor costs need to be considered. With technological updates and limitations solved, the detection of circulating lncRNAs applied in routine clinical practice will gain increasing popularity in the near future.

Conventional treatment strategies for BC are based on molecular subtypes, including luminal A, luminal B, HER2 type normal-like and basal-like [206]. However, BC is regarded as a highly heterogeneous disease, and it is urgent to find more individual and precise targets for personalized therapy to overcome the challenge of treatment resistance. The evidence we summarized above shows that chemoresistance-related lncRNAs may become potential targets for reversing resistance in theory. To date, there are several methods to change the expression of lncRNAs, such as siRNAs [207], antisense oligonucleotides (ASOs) [208] and clustered regularly interspaced short palindromic repeats-associated nuclease-9 (CRISPR/Cas9) systems [50]. Additionally, some small molecules are designed to regulate lncRNAs. For example, Hao et al. designed and identified a curcumin analogue named Comp34, which can inhibit BC by suppressing the oncogenic lncRNA *NUDT3-AS4* [209]. These preclinical studies showed evidence to support the ability to reverse therapeutic tolerance by targeting lncRNAs. However, due to the complex regulatory network and the safety of targeted therapies, it still faces great challenges to translate these methods into the clinic.

## 8. Conclusions and Perspectives

**Figure 8:**
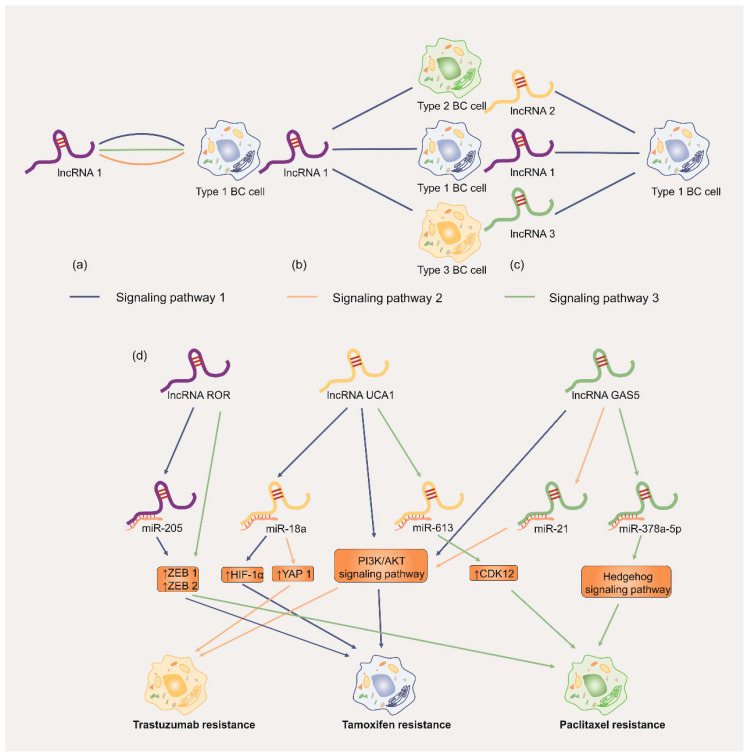
Brief sketch map of our conclusions in this review: (**a**) A certain lncRNA regulates chemoresistance in a subtype of BC cell via various signaling pathways; (**b**) a certain lncRNA induces different subtypes of BC cells to resist chemotherapeutic agents via the same signaling pathway; (**c**) a certain subtype of BC cell is regulated by various lncRNAs via the same signaling pathway; (**d**) the lncRNAs *UCA1*, *ROR*, and *GAS5* are used as examples to provide a further detailed explanation.

With advances in high-throughput sequencing technologies and bioinformatic analysis, the field of chemoresistance-related lncRNAs has attracted the interest of many investigators. In this review, we systematically summarized the mechanisms of MDR and the functions of lncRNAs mediated in chemoresistant BC, including apoptosis, autophagy, DNA repair, cell cycle, drug efflux metabolism, EMT, epigenetic modification and the TME. Moreover, the association of lncRNAs and immunotherapy are also briefly discussed. We predict the future prospects of using lncRNAs as early diagnostic and/or prognostic biomarkers and potential therapeutic targets for chemoresistant BC. Notably, an increasing number of studies have demonstrated that aberrant expression of lncRNAs in BC tissues is involved in MDR by regulating some intermediate regulatory pathways, which contributes to a better understanding of the molecular mechanisms of chemotherapeutic resistance. As discussed above, it can be concluded that certain lncRNAs can regulate MDR via various signalling pathways, certain lncRNAs can induce different subtypes of cells to resist chemotherapeutic agents via the same mechanism, and a subtype of BC cells can be associated with several lncRNAs in chemotherapeutic resistance (Figure 8). Each mechanism or pathway is not independent but interacts with others. In chemotherapy-resistant BC, the extensive crosstalk among lncRNA-mediated signalling pathways leads to the formation of complex networks. Therefore, advances in the research field of lncRNAs will be important to clarify their potential significance in chemoresistant BC. Furthermore, future cancer treatment strategies may improve the prognosis of patients by combining existing anticancer drugs with drugs targeting chemoresistance-related lncRNAs. Overall, a comprehensive, in-depth and thorough understanding of the mechanisms of lncRNA-mediated chemoresistance in BC is critical for reasonable innovation, rational design and successful translation of novel anticancer approaches to precision medicine with substantially improved clinical outcomes.

## Figures and Tables

**Table 1:** The role of lncRNAs in regulating cell survival and death in chemoresistant breast cancers.

Function	LncRNA	Type	Genomic Location	Expression Level *	Resistant Drugs	Cell Lines	Possible Mechanism ^§^	References
Suppressing apoptosis	*GAS5*	Tumor suppressor	chr1q25.1	↓	paclitaxel; cisplatin	MDA-MB-231; BT549	↑ miR-378a-5p/↓ SUFU signaling	[49]
	*MEG3*	Tumor suppressor	chr14q32	↓	doxorubicin; paclitaxel	Hs578T; MCF-7; MDA-MB-231	↑ TGF-β and N-cadherin protein; ↓ MMP 2, ZEB 1 and COL3A1 expression; ↓ miR-4513/↑ PBLD axis	[50,51]
	*PTENP1*	Tumor suppressor	N/A °	↓	adriamycin	MDA-MB-231; T-47D; MCF-7	↑ miR-20a/↓ PTEN axis; ↑ PI3K/AKT pathway	[52]
	*UCA1*	Oncogene	chr19q13.12	↑	tamoxifen	MCF-7; T-47D; LCC2; LCC9	↑ EZH2/↓ p21 axis; ↑ PI3K/AKT pathway; ↑ mTOR pathway	[53,54]
	*H19*	Oncogene	chr11p15.5	↑	paclitaxel	MDA-MB-453; MDA-MB-157; MDA-MB-231; ZR-75-1; MCF-7	↑ AKT pathway; ↓ BIK; ↓ NOXA	[47,48]
	*PRLB*	Oncogene	chr8p11.21	↑	5-fluorouracil	MDA-MB-231	↓ miR-4766-5p/↑ SIRT1 axis	[55]
	*LINP1*	Oncogene	chr10	↑	doxorubicin; 5-fluorouracil	MDA-MB-231; MDA-MB-468; MCF-7	↓ p53; ↓ E-cadherin; ↑ N-cadherin; ↑ vimentin; ↓ caspase9/Bax	[56]
	*LOC645166*	Oncogene	N/A	↑	adriamycin	MDA-MB-231; MCF-7	↑ NF-κB/GATA3 axis	[57]
Autophagy	*EGOT*	Tumor suppressor	N/A	↓	paclitaxel	MCF-7; T-47D; UACC-812; SK-BR-3; HCC70; MDA-MB-453; MDA-MB-231; MDA-MB-468; BT549; Hs578T	↑ ITPR1	[58]
	*ROR*	Oncogene	chr18q21.31	↑	tamoxifen	BT474	↑ MDR1 and GST-π mRNA; ↓ LC3 and Beclin 1	[59]
	*H19*	Oncogene	chr11p15.5	↑	tamoxifen	MCF-7	H19/SAHH/DNMT3B axis; ↑ Beclin1	[60]
	*ZNF649-AS1*	Oncogene	chr19q13.41	↑	trastuzumab	SK-BR-3; BT474	↑ ATG5 through associating with PTBP1	[61]
	*ASAH2B-2*	Oncogene	N/A	↑	everolimus	BT474; MCF-7	↑ mTOR pathway	[62]
DNA repair	*HCP5*	Oncogene	N/A	↑	cisplatin	MDA-MB-231	↓ PTEN	[63]
	*PTENP1*	Tumor suppressor	N/A	↓	adriamycin	MDA-MB-231; T-47D; MCF-7	↑ miR-20a/↓ PTEN axis; ↑ PI3K/AKT pathway	[52]
	*GAS5*	Tumor suppressor	chr1q25.1	↓	tamoxifen	MCF-7	↑ AKT/mTOR pathway; ↓ PTEN	[64]
	*UCA1*	Oncogene	chr19q13.12	↑	trastuzumab	SKBR-3	↓ miR-18a/↑ Yes-associated protein 1 (YAP1); ↓ PTEN; ↑ CD6	[65]
	*UCA1*	Oncogene	chr19q13.12	↑	paclitaxel	MCF-7	↓ miR-613/↑ CDK12 axis	[66]
	*GAS5*	Tumor suppressor	chr1q25.1	↓	trastuzumab; lapatinib	SKBR-3	↑ miR-21; ↓ PTEN; ↑ mTOR; ↑ Ki-67	[67]
	*LINC-PINT*	Tumor suppressor	N/A	↓	paclitaxel	MDA-MB-231; BT-20	↑ NONO	[68]
	*H19*	Oncogene	chr11p15.5	↑	doxorubicin	MCF-7	↓ PARP1	[69]
	*lncMat2B*	Oncogene	N/A	↑	cisplatin	MDA-MB-231; MCF-7	N/A	[70]
	*ADAMTS9-AS2*	Tumor suppressor	N/A	↓	tamoxifen	MCF-7	↑ microRNA-130a-5p; ↓ PTEN	[71]

* The expression in resistant BC lines is indicated by arrows; ↑ for higher expression and ↓ for lower expression. **^§^** The effect of lncRNAs on associated pathways, miRNAs, genes, or transcription factors involved in resistance mechanisms are indicated by arrows: ↑ induction and ↓ repression. ° N/A, information not available.

**Table 2:** The function of lncRNAs in chemoresistant breast cancers, including regulating cell cycle, drug efflux metabolism, EMT, and epigenetic alteration.

Function	LncRNA	Type	Genomic Location	Expression Level *	Resistant Drugs	Cell Lines	Possible Mechanism ^§^	References
regulating cell cycle	*TMPO-AS1*	Oncogene	N/A °	↑	tamoxifen	MCF-7	stabilize ESR1 mRNA	[109]
	*CASC2*	Oncogene	N/A	↑	paclitaxel	MDA-MB-231; MCF-7	↓ miR-18a-5p/↑ CDK19 axis	[110]
	*LINC00511*	Oncogene	chr17q24.3	↑	paclitaxel	MDA-MB-231; MCF-7; T-47D; Hs-578T	↓ miR-29c/↑ CDK6 axis	[104]
	*NEAT1*	Oncogene	N/A	↑	cisplatin/taxol	MDA-MB-231	N/A	[111]
	*LOL*	Oncogene	N/A	↑	tamoxifen	MCF-7	↓ let-7 miRNA; ↓ ERα signaling	[112]
	*UCA1*	Oncogene	chr19q13.12	↑	tamoxifen	MCF-7; T-47D; LCC2; LCC9; BT474	↑ EZH2/↓ p21 axis; ↑ PI3K/AKT pathway; ↓ miR-18a/↑ HIF1α	[54,113]
	*DSCAM-AS1*	Oncogene	chr21q22.3	↑	tamoxifen	MCF-7; T-47D; SK-BR-3; MDA-MB-231	↑ epidermal growth factor receptor pathway substrate 8 (EPS8); ↑ ESR1; ↑ ERα; ↓ miR-137	[114,115]
	*FTH1P3*	Oncogene	N/A	↑	paclitaxel	MCF-7; MDA-MB-231; MDA-MB-468; MDA-MB-453	↓ miR-206/↑ ABCB1	[116]
	*MAFG-AS1*	Oncogene	N/A	↑	tamoxifen	MCF-7; BT474; T-47D; MCF10A	↓ miR-339-5p/↑ CDK2 axis	[117]
	*PRLB*	Oncogene	chr8p11.21	↑	5-fluorouracil	MDA-MB-231	↓ miR-4766-5p/↑ SIRT1 axis	[55]
	*UCA1*	Oncogene	chr19q13.12	↑	trastuzumab	SKBR-3	↓ miR-18a/↑ Yes-associated protein 1 (YAP1); ↓ PTEN; ↑ CD6	[65]
	*LINP1*	Oncogene	chr10	↑	doxorubicin; 5-fluorouracil	MDA-MB-231; MDA-MB-468; MCF-7	↓ p53; ↓ E-cadherin; ↑ N-cadherin; ↑ vimentin; ↓ caspase9/Bax	[56]
	*TROJAN*	Oncogene	N/A	↑	palbociclib	MCF7; T47D	↑ NKRF/CDK2 axis	[5]
	*DILA1*	Oncogene	N/A	↑	tamoxifen	MCF-7; 293-T; T47D	↑ Cyclin D1	[4]
	*ARA*	Oncogene	Xq23	↑	adriamycin	MCF-7	multiple signaling pathways	[118]
drug efflux metabolism	*GAS5*	Tumor suppressor	chr1q25.1	↓	adriamycin	MCF-7	↑ miR-221-3p/↑ Dickkopf 2 (DKK2) axis; ↑ Wnt/b-catenin pathway	[119]
	*BC032585*	Tumor suppressor	chr9	↓	taxane; anthracyclines	MDA-MB-231	↑ MDR1	[120]
	*Linc00518*	Oncogene	chr6	↑	multidrug adriamycin; vincristine; paclitaxel	MCF-7	↓ miR-199a/↑ MRP1 axis	[121]
	*FTH1P3*	Oncogene	N/A	↑	paclitaxel	MCF-7; MDA-MB-231; MDA-MB-468; MDA-MB-453	↓ miR-206/↑ ABCB1	[116]
	*ROR*	Oncogene	chr18q21.31	↑	tamoxifen	BT474	↑ MDR1 and GST-π mRNA; ↓ LC3 and Beclin 1	[59]
	*H19*	Oncogene	chr11p15.5	↑	doxorubicin; anthracyclines	MCF-7	↑ CUL4A-ABCB1/MDR1 pathway	[122]
	*RP11-770J1.3* *TMEM25*	Oncogene	N/A	↑	paclitaxel	MCF-7	↑ MRP, BCRP and MDR1/P-gp	[123]
EMT	*LINP1*	Oncogene	chr10	↑	tamoxifen	MCF-7; T-47D	↓ ER expression signaling pathway	[124]
	*MEG3*	Tumor suppressor	chr14q32	↓	doxorubicin	Hs578T	↑ TGF-β and N-cadherin protein; ↓ MMP 2, ZEB 1 and COL3A1 expression	[50]
	*NONHSAT101069*	Oncogene	chr5	↑	epirubicin	MCF-7	↓ miR-129-5p/↑ Twist1 axis	[125]
	*NEAT1*	Oncogene	N/A	↑	cisplatin/taxol	MDA-MB-231	N/A	[111]
	*H19*	Oncogene	chr11p15.5	↑	tamoxifen; paclitaxel	SK-BR-3; MCF-7	↑ Wnt pathway; ↓ miR-340-3p/YWHAZ axis	[126,127]
	*PRLB*	Oncogene	chr8p11.21	↑	5-fluorouracil	MDA-MB-231	↓ miR-4766-5p/↑ SIRT1	[55]
	*LINC00894002*	Tumor suppressor	X chromosome	↓	tamoxifen	MCF-7	↓ miR200/↑ TGFβ2 signaling pathway; ↑ ZEB1	[128]
	*LINP1*	Oncogene	chr10	↑	doxorubicin; 5-fluorouracil	MDA-MB-231; MDA-MB-468; MCF-7	↓ p53; ↓ E-cadherin; ↑ N-cadherin; ↑ vimentin; ↓ caspase9/Bax	[56]
	*NEAT1*	Oncogene	N/A	↑	5-fluorouracil	MCF-7; T-47D; MDA-MB-231; ZR-75-1	↓ miR-211/↑ HMGA2 axis	[129]
	*ROR*	Oncogene	chr18q21.31	↑	tamoxifen	MDA-MB-231; MCF-7	↓ microRNA-205; ↓ E-cadherin; ↑ vimentin; ↑ ZEB1 and ZEB2	[130]
	*DLX6-AS1*	Oncogene	N/A	↑	cisplatin	HCC1599; MDA-MB-231; HCC1806; Hs578T	↓ miR-199b-5p/paxillin signaling	[131]
	*ROR*	Oncogene	chr18q21.31	↑	5-fluorouracil; paclitaxel	T-47D; MCF-7; SK-BR-3; Bcap-37; MDA-MB-231; MCF10A	↓ E-cadherin; ↑ vimentin and N-cadherin	[132]
	*ATB*	Oncogene	chr14q11.2	↑	trastuzumab	SKBR-3	↓ miR-200c; ↑ TGF-β signaling; ↑ ZEB1 and ZNF-217	[133]
	*SNHG7*	Oncogene	chr9q34.3	↑	trastuzumab; adriamycin; paclitaxel	SKBR3; AU565; MDA-MB-231; MCF10A; MCF-7	↓ miR-186; ↓ miR-34a	[134,135]
	*DCST1-AS1*	Oncogene	N/A	↑	doxorubicin; paclitaxel	MDA-MB-231; BT-549; T-47D; MCF-7	↑ TGF-β/Smad signaling through ANXA1	[136]
epigenetic alteration	*LINC00969*	Oncogene	N/A	↑	trastuzumab	SKBR-3; BT474	↑ translation and stability of ERBB2 mRNA	[137]
	*TMPO-AS1*	Oncogene	N/A	↑	tamoxifen	MCF-7	stabilize ESR1 mRNA	[109]
	*ZNF649-AS1*	Oncogene	chr19q13.41	↑	trastuzumab	SK-BR-3; BT474	↑ ATG5 through associating with PTBP1	[61]
	*MIR2052HG*	Oncogene	N/A	↑	aromatase inhibitor	MDA-MB-231; CAMA-1; Au565; 293-T; MCF-7	↑ LMTK3; ↓ AKT/FOXO3-mediated ESR1 transcription; ↓ PKC/MEK/ERK/RSK1 pathway; ↓ ERα degradation	[138]
	*LINC00472*	Tumor suppressor	N/A	↓	tamoxifen	MCF-7; T-47D; MDA-MB-231; Hs578T	↑ phosphorylation NF-κB	[139]
	*UCA1*	Oncogene	chr19q13.12	↑	tamoxifen	MCF-7; T-47D; LCC2; LCC9	↑ EZH2/↓ p21 axis; ↑ PI3K/AKT pathway	[54]
	*H19*	Oncogene	chr11p15.5	↑	tamoxifen; fulverstrant	LCC2; LCC9; MCF-7	↑ ERα; ↑ Notch, HGF and c-MET signaling	[140]
	*BORG*	Oncogene	N/A	↑	doxorubicin	D2.OR; 67NR; 4T07; 4T1	↑ NF-κB signaling; ↑ RPA1	[141]
	*SNHG14*	Oncogene	chr15q11.2	↑	trastuzumab	SKBR-3; BT474	↑ PABPC1; ↑ Nrf2 pathway	[142]
	*MAPT-AS1*	Oncogene	chr17q21.31	↑	paclitaxel	MDA-MB-231; MDA-MB-468	↑ MAPT mRNA	[143]
	*Linc-RoR*	Oncogene	N/A	↑	tamoxifen	MCF-7	↑ MAPK/ERK signaling; ↑ ER signaling; ↓ DUSP7	[144]
	*HOTAIR*	Oncogene	chr12q13.13	↑	tamoxifen; TNF-a	MCF-7; T-47D	↑ ER signaling; ↑ SRC and p38MAPK kinases; ↑ EZH2	[145,146]
	*H19*	Oncogene	chr11p15.5	↑	paclitaxel	ZR-75-1; MCF-7	↓ BIK; ↓ NOXA	[48]
	*BDNF-AS*	Oncogene	chr11p14.1	↑	tamoxifen	MCF-7; T-47D; MDA-MB-231	↑ RNH1/TRIM21/mTOR	[147]
	*BCAR4*	Oncogene	chr16p13.13	↑	tamoxifen	ZR-75-1	↑ ERBB2/ERBB3 pathway; ↑ AKT	[148]

* The expression in resistant BC lines is indicated by arrows: ↑ for higher expression and ↓ for lower expression. **^§^** The effect of lncRNAs on associated pathways, miRNAs, genes, or transcription factors involved in resistance mechanisms are indicated by arrows: ↑ induction and ↓ repression. ° N/A, information not available.

## References

[1] Loibl S., Poortmans P., Morrow M., Denkert C., Curigliano G. (2021). Breast cancer. Lancet.

[2] Nikolaou M., Pavlopoulou A., Georgakilas A.G., Kyrodimos E. (2018). The challenge of drug resistance in cancer treatment: A current overview. Clin. Exp. Metastasis.

[3] Qian X., Zhao J., Yeung P.Y., Zhang Q.C., Kwok C.K. (2019). Revealing lncRNA Structures and Interactions by Sequencing-Based Approaches. Trends. Biochem. Sci..

[4] Shi Q., Li Y., Li S., Jin L., Lai H., Wu Y., Cai Z., Zhu M., Li Q., Li Y. (2020). LncRNA DILA1 inhibits Cyclin D1 degradation and contributes to tamoxifen resistance in breast cancer. Nat. Commun..

[5] Jin X., Ge L.-P., Li D.-Q., Shao Z.-M., Di G.-H., Xu X.-E., Jiang Y.-Z. (2020). LncRNA TROJAN promotes proliferation and resistance to CDK4/6 inhibitor via CDK2 transcriptional activation in ER+ breast cancer. Mol. Cancer.

[6] Wu Q., Yang Z., Nie Y., Shi Y., Fan D. (2014). Multi-drug resistance in cancer chemotherapeutics: Mechanisms and lab approaches. Cancer Lett..

[7] Giaccone G., Pinedo H.M. (1996). Drug Resistance. Oncologist.

[8] Li W., Zhang H., Assaraf Y.G., Zhao K., Xu X., Xie J., Yang D.-H., Chen Z.-S. (2016). Overcoming ABC transporter-mediated multidrug resistance: Molecular mechanisms and novel therapeutic drug strategies. Drug Resist. Updates Rev. Comment. Antimicrob. Anticancer Chemother..

[9] Kapse-Mistry S., Govender T., Srivastava R., Yergeri M. (2014). Nanodrug delivery in reversing multidrug resistance in cancer cells. Front. Pharmacol..

[10] Perez J., Bardin C., Rigal C., Anthony B., Rousseau R., Dutour A. (2011). Anti-MDR1 siRNA restores chemosensitivity in chemoresistant breast carcinoma and osteosarcoma cell lines. Anticancer Res..

[11] Mechetner E.B., Roninson I.B. (1992). Efficient inhibition of P-glycoprotein-mediated multidrug resistance with a monoclonal antibody. Proc. Natl. Acad. Sci. USA.

[12] Kartal-Yandim M., Adan-Gokbulut A., Baran Y. (2016). Molecular mechanisms of drug resistance and its reversal in cancer. Crit. Rev. Biotechnol..

[13] Koski A., Raki M., Nokisalmi P., Liikanen I., Kangasniemi L., Joensuu T., Kanerva A., Pesonen S., Alemany R., Hemminki A. (2012). Verapamil results in increased blood levels of oncolytic adenovirus in treatment of patients with advanced cancer. Mol. Ther. J. Am. Soc. Gene Ther..

[14] Assaraf Y.G., Brozovic A., Gonçalves A.C., Jurkovicova D., Linē A., Machuqueiro M., Saponara S., Sarmento-Ribeiro A.B., Xavier C.P.R., Vasconcelos M.H. (2019). The multi-factorial nature of clinical multidrug resistance in cancer. Drug Resist. Updates Rev. Comment. Antimicrob. Anticancer Chemother..

[15] Leonard G.D., Fojo T., Bates S.E. (2003). The role of ABC transporters in clinical practice. Oncologist.

[16] Lin W., Zhou Q., Wang C.-Q., Zhu L., Bi C., Zhang S., Wang X., Jin H. (2020). LncRNAs regulate metabolism in cancer. Int. J. Biol. Sci..

[17] Bridges M.C., Daulagala A.C., Kourtidis A. (2021). LNCcation: lncRNA localization and function. J. Cell Biol..

[18] Peng W.X., Koirala P., Mo Y.Y. (2017). LncRNA-mediated regulation of cell signaling in cancer. Oncogene.

[19] Tam C., Wong J.H., Tsui S.K.W., Zuo T., Chan T.F., Ng T.B. (2019). LncRNAs with miRNAs in regulation of gastric, liver, and colorectal cancers: Updates in recent years. Appl. Microbiol. Biotechnol..

[20] Sartori D.A., Chan D.W. (2014). Biomarkers in prostate cancer: What’s new?. Curr. Opin. Oncol..

[21] Xue W.-J., Ying X.-L., Jiang J.-H., Xu Y.-H. (2014). Prostate cancer antigen 3 as a biomarker in the urine for prostate cancer diagnosis: A meta-analysis. J. Cancer Res. Ther.

[22] Hu B., Yang H., Yang H. (2014). Diagnostic value of urine prostate cancer antigen 3 test using a cutoff value of 35 μg/L in patients with prostate cancer. Tumour Biol. J. Int. Soc. Oncodev. Biol. Med..

[23] Rinn J.L., Chang H.Y. (2012). Genome regulation by long noncoding RNAs. Annu. Rev. Biochem..

[24] Arita T., Ichikawa D., Konishi H., Komatsu S., Shiozaki A., Shoda K., Kawaguchi T., Hirajima S., Nagata H., Kubota T. (2013). Circulating long non-coding RNAs in plasma of patients with gastric cancer. Anticancer Res..

[25] Yuan S., Xiang Y., Guo X., Zhang Y., Li C., Xie W., Wu N., Wu L., Cai T., Ma X. (2020). Circulating Long Noncoding RNAs Act as Diagnostic Biomarkers in Non-Small Cell Lung Cancer. Front. Oncol.

[26] Tang H., Wu Z., Zhang J., Su B. (2013). Salivary lncRNA as a potential marker for oral squamous cell carcinoma diagnosis. Mol. Med. Rep..

[27] Bolha L., Ravnik-Glavač M., Glavač D. (2017). Long Noncoding RNAs as Biomarkers in Cancer. Dis. Markers.

[28] Zhang K., Shi H., Xi H., Wu X., Cui J., Gao Y., Liang W., Hu C., Liu Y., Li J. (2017). Genome-Wide lncRNA Microarray Profiling Identifies Novel Circulating lncRNAs for Detection of Gastric Cancer. Theranostics.

[29] Lu L., Li J., Moussaoui M., Boix E. (2018). Immune Modulation by Human Secreted RNases at the Extracellular Space. Front. Immunol..

[30] Clark M.B., Johnston R.L., Inostroza-Ponta M., Fox A.H., Fortini E., Moscato P., Dinger M.E., Mattick J.S. (2012). Genome-wide analysis of long noncoding RNA stability. Genome Res..

[31] Giraldez M.D., Spengler R.M., Etheridge A., Goicochea A.J., Tuck M., Choi S.W., Galas D.J., Tewari M. (2019). Phospho-RNA-seq: A modified small RNA-seq method that reveals circulating mRNA and lncRNA fragments as potential biomarkers in human plasma. EMBO J..

[32] Charles Richard J.L., Eichhorn P.J.A. (2018). Platforms for Investigating LncRNA Functions. SLAS Technol..

[33] Galamb O., Barták B.K., Kalmár A., Nagy Z.B., Szigeti K.A., Tulassay Z., Igaz P., Molnár B. (2019). Diagnostic and prognostic potential of tissue and circulating long non-coding RNAs in colorectal tumors. World J. Gastroenterol..

[34] Long Y., Wang X., Youmans D.T., Cech T.R. (2017). How do lncRNAs regulate transcription?. Sci. Adv..

[35] Taft R.J., Pang K.C., Mercer T.R., Dinger M., Mattick J.S. (2010). Non-coding RNAs: Regulators of disease. J. Pathol..

[36] D’Arcy M.S. (2019). Cell death: A review of the major forms of apoptosis, necrosis and autophagy. Cell Biol. Int..

[37] Grilo A.L., Mantalaris A. (2019). Apoptosis: A mammalian cell bioprocessing perspective. Biotechnol. Adv..

[38] Pistritto G., Trisciuoglio D., Ceci C., Garufi A., D’Orazi G. (2016). Apoptosis as anticancer mechanism: Function and dysfunction of its modulators and targeted therapeutic strategies. Aging.

[39] Xu J.-H., Hu S.-L., Shen G.-D., Shen G. (2016). Tumor suppressor genes and their underlying interactions in paclitaxel resistance in cancer therapy. Cancer Cell Int..

[40] Yuan Z., Jiang H., Zhu X., Liu X., Li J. (2017). Ginsenoside Rg3 promotes cytotoxicity of Paclitaxel through inhibiting NF-κB signaling and regulating Bax/Bcl-2 expression on triple-negative breast cancer. Biomed. Pharmacother..

[41] Ma Y., Zhou G., Li M., Hu D., Zhang L., Liu P., Lin K. (2018). Long noncoding RNA DANCR mediates cisplatin resistance in glioma cells via activating AXL/PI3K/Akt/NF-κB signaling pathway. Neurochem. Int..

[42] Sun Z.-Y., Jian Y.-K., Zhu H.-Y., Li B. (2019). lncRNAPVT1 targets miR-152 to enhance chemoresistance of osteosarcoma to gemcitabine through activating c-MET/PI3K/AKT pathway. Pathol. Res. Pract..

[43] Wang H., Fang L., Jiang J., Kuang Y., Wang B., Shang X., Han P., Li Y., Liu M., Zhang Z. (2018). The cisplatin-induced lncRNA PANDAR dictates the chemoresistance of ovarian cancer via regulating SFRS2-mediated p53 phosphorylation. Cell Death Dis..

[44] Sun Y., Hu B., Wang Q., Ye M., Qiu Q., Zhou Y., Zeng F., Zhang X., Guo Y., Guo L. (2018). Long non-coding RNA HOTTIP promotes BCL-2 expression and induces chemoresistance in small cell lung cancer by sponging miR-216a. Cell Death Dis..

[45] Collette J., Le Bourhis X., Adriaenssens E. (2017). Regulation of Human Breast Cancer by the Long Non-Coding RNA H19. Int. J. Mol. Sci..

[46] Li Y., Ma H.-Y., Hu X.-W., Qu Y.-Y., Wen X., Zhang Y., Xu Q.-Y. (2020). LncRNA H19 promotes triple-negative breast cancer cells invasion and metastasis through the p53/TNFAIP8 pathway. Cancer Cell Int..

[47] Han J., Han B., Wu X., Hao J., Dong X., Shen Q., Pang H. (2018). Knockdown of lncRNA H19 restores chemo-sensitivity in paclitaxel-resistant triple-negative breast cancer through triggering apoptosis and regulating Akt signaling pathway. Toxicol. Appl. Pharmacol..

[48] Si X., Zang R., Zhang E., Liu Y., Shi X., Zhang E., Shao L., Li A., Yang N., Han X. (2016). LncRNA H19 confers chemoresistance in ERα-positive breast cancer through epigenetic silencing of the pro-apoptotic gene BIK. Oncotarget.

[49] Zheng S., Li M., Miao K., Xu H. (2020). lncRNA GAS5-promoted apoptosis in triple-negative breast cancer by targeting miR-378a-5p/SUFU signaling. J. Cell. Biochem..

[50] Deocesano-Pereira C., Machado R.A.C., De Jesus-Ferreira H.C., Marchini T., Pereira T.F., Carreira A.C.O., Sogayar M.C. (2019). Functional impact of the long non-coding RNA deletion by CRISPR/Cas9 in the human triple negative metastatic Hs578T cancer cell line. Oncol. Lett..

[51] Zhu M., Wang F., Mi H., Li L., Wang J., Han M., Gu Y. (2020). Long noncoding RNA MEG3 suppresses cell proliferation, migration and invasion, induces apoptosis and paclitaxel-resistance via miR-4513/PBLD axis in breast cancer cells. Cell Cycle.

[52] Gao X., Qin T., Mao J., Zhang J., Fan S., Lu Y., Sun Z., Zhang Q., Song B., Li L. (2019). PTENP1/miR-20a/PTEN axis contributes to breast cancer progression by regulating PTEN via PI3K/AKT pathway. J. Exp. Clin. Cancer Res..

[53] Wu C., Luo J. (2016). Long Non-Coding RNA (lncRNA) Urothelial Carcinoma-Associated 1 (UCA1) Enhances Tamoxifen Resistance in Breast Cancer Cells via Inhibiting mTOR Signaling Pathway. Med. Sci. Monit..

[54] Li Z., Yu D., Li H., Lv Y., Li S. (2019). Long non-coding RNA UCA1 confers tamoxifen resistance in breast cancer endocrinotherapy through regulation of the EZH2/p21 axis and the PI3K/AKT signaling pathway. Int. J. Oncol..

[55] Liang Y., Song X., Li Y., Sang Y., Zhang N., Zhang H., Liu Y., Duan Y., Chen B., Guo R. (2018). A novel long non-coding RNA-PRLB acts as a tumor promoter through regulating miR-4766-5p/SIRT1 axis in breast cancer. Cell. Death Dis..

[56] Liang Y., Li Y., Song X., Zhang N., Sang Y., Zhang H., Liu Y., Chen B., Zhao W., Wang L. (2018). Long noncoding RNA LINP1 acts as an oncogene and promotes chemoresistance in breast cancer. Cancer Biol. Ther..

[57] Zheng R., Jia J., Guan L., Yuan H., Liu K., Liu C., Ye W., Liao Y., Lin S., Huang O. (2020). Long noncoding RNA lnc-LOC645166 promotes adriamycin resistance via NF-κB/GATA3 axis in breast cancer. Aging.

[58] Xu S., Wang P., Zhang J., Wu H., Sui S., Zhang J., Wang Q., Qiao K., Yang W., Xu H. (2019). Ai-lncRNA EGOT enhancing autophagy sensitizes paclitaxel cytotoxicity via upregulation of ITPR1 expression by RNA-RNA and RNA-protein interactions in human cancer. Mol. Cancer.

[59] Li Y., Jiang B., Zhu H., Qu X., Zhao L., Tan Y., Jiang Y., Liao M., Wu X. (2017). Inhibition of long non-coding RNA ROR reverses resistance to Tamoxifen by inducing autophagy in breast cancer. Tumour Biol..

[60] Wang J., Xie S., Yang J., Xiong H., Jia Y., Zhou Y., Chen Y., Ying X., Chen C., Ye C. (2019). The long noncoding RNA H19 promotes tamoxifen resistance in breast cancer via autophagy. J. Hematol. Oncol..

[61] Han M., Qian X., Cao H., Wang F., Li X., Han N., Yang X., Yang Y., Dou D., Hu J. (2020). lncRNA ZNF649-AS1 Induces Trastuzumab Resistance by Promoting ATG5 Expression and Autophagy. Mol. Ther. J. Am. Soc. Gene Ther..

[62] Li J., Zhang J., Jin L., Deng H., Wu J. (2018). Silencing Inhibits Breast Cancer Cell Growth the mTOR Pathway. Anticancer Res..

[63] Wu J., Chen H., Ye M., Wang B., Zhang Y., Sheng J., Meng T., Chen H. (2019). Downregulation of long noncoding RNA HCP5 contributes to cisplatin resistance in human triple-negative breast cancer via regulation of PTEN expression. Biomed. Pharmacother..

[64] Jiang Y., Qian T., Li S., Xie Y., Tao M. (2022). Metformin reverses tamoxifen resistance through the lncRNA GAS5-medicated mTOR pathway in breast cancer. Ann. Transl. Med..

[65] Zhu H.-Y., Bai W.-D., Ye X.-M., Yang A.-G., Jia L.-T. (2018). Long non-coding RNA UCA1 desensitizes breast cancer cells to trastuzumab by impeding miR-18a repression of Yes-associated protein 1. Biochem. Biophys. Res. Commun..

[66] Liu C., Jiang F., Zhang X., Xu X. (2020). Long Non-Coding RNA UCA1 Modulates Paclitaxel Resistance in Breast Cancer via miR-613/CDK12 Axis. Cancer Manag. Res..

[67] Li W., Zhai L., Wang H., Liu C., Zhang J., Chen W., Wei Q. (2016). Downregulation of LncRNA GAS5 causes trastuzumab resistance in breast cancer. Oncotarget.

[68] Chen J., Zhu M., Zou L., Xia J., Huang J., Deng Q., Xu R. (2020). Long non-coding RNA LINC-PINT attenuates paclitaxel resistance in triple-negative breast cancer cells via targeting the RNA-binding protein NONO. Acta Biochim. Biophys. Sin..

[69] Wang Y., Zhou P., Li P., Yang F., Gao X.-Q. (2020). Long non-coding RNA H19 regulates proliferation and doxorubicin resistance in MCF-7 cells by targeting PARP1. Bioengineered.

[70] García-Venzor A., Mandujano-Tinoco E.A., Ruiz-Silvestre A., Sánchez J.M., Lizarraga F., Zampedri C., Melendez-Zajgla J., Maldonado V. (2020). lncMat2B regulated by severe hypoxia induces cisplatin resistance by increasing DNA damage repair and tumor-initiating population in breast cancer cells. Carcinogenesis.

[71] Shi Y.F., Lu H., Wang H.B. (2019). Downregulated lncRNA ADAMTS9-AS2 in breast cancer enhances tamoxifen resistance by activating microRNA-130a-5p. Eur. Rev. Med. Pharmacol. Sci..

[72] Mizushima N., Komatsu M. (2011). Autophagy: Renovation of cells and tissues. Cell.

[73] Mizushima N., Levine B., Cuervo A.M., Klionsky D.J. (2008). Autophagy fights disease through cellular self-digestion. Nature.

[74] Shintani T., Klionsky D.J. (2004). Autophagy in health and disease: A double-edged sword. Science.

[75] Maiuri M.C., Zalckvar E., Kimchi A., Kroemer G. (2007). Self-eating and self-killing: Crosstalk between autophagy and apoptosis. Nat. Rev. Mol. Cell Biol..

[76] Ravegnini G., Sammarini G., Nannini M., Pantaleo M.A., Biasco G., Hrelia P., Angelini S. (2017). Gastrointestinal stromal tumors (GIST): Facing cell death between autophagy and apoptosis. Autophagy.

[77] John S., Nayvelt I., Hsu H.-C., Yang P., Liu W., Das G.M., Thomas T., Thomas T.J. (2008). Regulation of estrogenic effects by beclin 1 in breast cancer cells. Cancer Res..

[78] Jackson S.P., Bartek J. (2009). The DNA-damage response in human biology and disease. Nature.

[79] Palchaudhuri R., Hergenrother P.J. (2007). DNA as a target for anticancer compounds: Methods to determine the mode of binding and the mechanism of action. Curr. Opin. Biotechnol..

[80] Nikitaki Z., Michalopoulos I., Georgakilas A.G. (2015). Molecular inhibitors of DNA repair: Searching for the ultimate tumor killing weapon. Future Med. Chem..

[81] Du P., Zhao H., Peng R., Liu Q., Yuan J., Peng G., Liao Y. (2017). LncRNA-XIST interacts with to modulate the chemoresistance of glioma cell to TMZ through DNA mismatch repair pathway. Biosci. Rep..

[82] Wang X., Liu H., Shi L., Yu X., Gu Y., Sun X. (2018). LINP1 facilitates DNA damage repair through non-homologous end joining (NHEJ) pathway and subsequently decreases the sensitivity of cervical cancer cells to ionizing radiation. Cell Cycle.

[83] Chen C.-C., Chen C.-Y., Wang S.-H., Yeh C.-T., Su S.-C., Ueng S.-H., Chuang W.-Y., Hsueh C., Wang T.-H. (2018). Melatonin Sensitizes Hepatocellular Carcinoma Cells to Chemotherapy Through Long Non-Coding RNA RAD51-AS1-Mediated Suppression of DNA Repair. Cancers.

[84] Shen W.H., Balajee A.S., Wang J., Wu H., Eng C., Pandolfi P.P., Yin Y. (2007). Essential role for nuclear PTEN in maintaining chromosomal integrity. Cell.

[85] Bassi C., Ho J., Srikumar T., Dowling R.J.O., Gorrini C., Miller S.J., Mak T.W., Neel B.G., Raught B., Stambolic V. (2013). Nuclear PTEN controls DNA repair and sensitivity to genotoxic stress. Science.

[86] Dillon L.M., Miller T.W. (2014). Therapeutic targeting of cancers with loss of PTEN function. Curr. Drug Targets.

[87] Li L., Ross A.H. (2007). Why is PTEN an important tumor suppressor?. J. Cell. Biochem..

[88] He J., Kang X., Yin Y., Chao K.S.C., Shen W.H. (2015). PTEN regulates DNA replication progression and stalled fork recovery. Nat. Commun..

[89] Feng J., Liang J., Li J., Li Y., Liang H., Zhao X., McNutt M.A., Yin Y. (2015). PTEN Controls the DNA Replication Process through MCM2 in Response to Replicative Stress. Cell Rep..

[90] Wang G., Li Y., Wang P., Liang H., Cui M., Zhu M., Guo L., Su Q., Sun Y., McNutt M.A. (2015). PTEN regulates RPA1 and protects DNA replication forks. Cell Res..

[91] van Ree J.H., Nam H.-J., Jeganathan K.B., Kanakkanthara A., van Deursen J.M. (2016). Pten regulates spindle pole movement through Dlg1-mediated recruitment of Eg5 to centrosomes. Nat. Cell Biol..

[92] He J., Zhang Z., Ouyang M., Yang F., Hao H., Lamb K.L., Yang J., Yin Y., Shen W.H. (2016). PTEN regulates EG5 to control spindle architecture and chromosome congression during mitosis. Nat. Commun..

[93] Zhang Z., Hou S.-Q., He J., Gu T., Yin Y., Shen W.H. (2016). PTEN regulates PLK1 and controls chromosomal stability during cell division. Cell Cycle.

[94] Sonnenblick A., de Azambuja E., Azim H.A., Piccart M. (2015). An update on PARP inhibitors-moving to the adjuvant setting. Nat. Rev. Clin. Oncol..

[95] Brandmaier A., Hou S.-Q., Shen W.H. (2017). Cell Cycle Control by PTEN. J. Mol. Biol..

[96] Coffman J.A. (2004). Cell cycle development. Dev. Cell.

[97] Malumbres M., Barbacid M. (2009). Cell cycle, CDKs and cancer: A changing paradigm. Nat. Rev. Cancer.

[98] Asghar U., Witkiewicz A.K., Turner N.C., Knudsen E.S. (2015). The history and future of targeting cyclin-dependent kinases in cancer therapy. Nat. Rev. Drug Discov..

[99] Otto T., Sicinski P. (2017). Cell cycle proteins as promising targets in cancer therapy. Nat. Rev. Cancer.

[100] Wang T.H., Wang H.S., Soong Y.K. (2000). Paclitaxel-induced cell death: Where the cell cycle and apoptosis come together. Cancer.

[101] Tishler R.B., Geard C.R., Hall E.J., Schiff P.B. (1992). Taxol sensitizes human astrocytoma cells to radiation. Cancer Res..

[102] Tishler R.B., Schiff P.B., Geard C.R., Hall E.J. (1992). Taxol: A novel radiation sensitizer. Int. J. Radiat. Oncol. Biol. Phys..

[103] Rowinsky E.K., Donehower R.C. (1995). Paclitaxel (taxol). N. Engl. J. Med..

[104] Zhang H., Zhao B., Wang X., Zhang F., Yu W. (2019). LINC00511 knockdown enhances paclitaxel cytotoxicity in breast cancer via regulating miR-29c/CDK6 axis. Life Sci..

[105] Rader J., Russell M.R., Hart L.S., Nakazawa M.S., Belcastro L.T., Martinez D., Li Y., Carpenter E.L., Attiyeh E.F., Diskin S.J. (2013). Dual CDK4/CDK6 inhibition induces cell-cycle arrest and senescence in neuroblastoma. Clin. Cancer Res. Off. J. Am. Assoc. Cancer Res..

[106] Wander S.A., Mayer E.L., Burstein H.J. (2017). Blocking the Cycle: Cyclin-Dependent Kinase 4/6 Inhibitors in Metastatic, Hormone Receptor-Positive Breast Cancer. J. Clin. Oncol. Off. J. Am. Soc. Clin. Oncol..

[107] Braal C.L., Jongbloed E.M., Wilting S.M., Mathijssen R.H.J., Koolen S.L.W., Jager A. (2021). Inhibiting CDK4/6 in Breast Cancer with Palbociclib, Ribociclib, and Abemaciclib: Similarities and Differences. Drugs.

[108] Piezzo M., Cocco S., Caputo R., Cianniello D., Gioia G.D., Lauro V.D., Fusco G., Martinelli C., Nuzzo F., Pensabene M. (2020). Targeting Cell Cycle in Breast Cancer: CDK4/6 Inhibitors. Int. J. Mol. Sci..

[109] Mitobe Y., Ikeda K., Suzuki T., Takagi K., Kawabata H., Horie-Inoue K., Inoue S. (2019). *ESR1*-Stabilizing Long Noncoding RNA Promotes Hormone-Refractory Breast Cancer Progression. Mol. Cell. Biol..

[110] Zheng P., Dong L., Zhang B., Dai J., Zhang Y., Wang Y., Qin S. (2019). Long noncoding RNA CASC2 promotes paclitaxel resistance in breast cancer through regulation of miR-18a-5p/CDK19. Histochem. Cell. Biol..

[111] Shin V.Y., Chen J., Cheuk I.W.-Y., Siu M.-T., Ho C.-W., Wang X., Jin H., Kwong A. (2019). Long non-coding RNA NEAT1 confers oncogenic role in triple-negative breast cancer through modulating chemoresistance and cancer stemness. Cell Death Dis..

[112] Sun W., Xu X., Jiang Y., Jin X., Zhou P., Liu Y., Guo Y., Ma D., Zuo W., Huang S. (2019). Transcriptome analysis of luminal breast cancer reveals a role for LOL in tumor progression and tamoxifen resistance. Int. J. Cancer.

[113] Li X., Wu Y., Liu A., Tang X. (2016). Long non-coding RNA UCA1 enhances tamoxifen resistance in breast cancer cells through a miR-18a-HIF1α feedback regulatory loop. Tumour Biol. J. Int. Soc. Oncodev. Biol. Med..

[114] Sun W., Li A.-Q., Zhou P., Jiang Y.-Z., Jin X., Liu Y.-R., Guo Y.-J., Yang W.-T., Shao Z.-M., Xu X.-E. (2018). DSCAM-AS1 regulates the G/S cell cycle transition and is an independent prognostic factor of poor survival in luminal breast cancer patients treated with endocrine therapy. Cancer Med..

[115] Ma Y., Bu D., Long J., Chai W., Dong J. (2019). LncRNA DSCAM-AS1 acts as a sponge of miR-137 to enhance Tamoxifen resistance in breast cancer. J. Cell. Physiol..

[116] Wang R., Zhang T., Yang Z., Jiang C., Seng J. (2018). Long non-coding RNA FTH1P3 activates paclitaxel resistance in breast cancer through miR-206/ABCB1. J. Cell. Mol. Med..

[117] Feng J., Wen T., Li Z., Feng L., Zhou L., Yang Z., Xu L., Shi S., Hou K., Shen J. (2020). Cross-talk between the ER pathway and the lncRNA MAFG-AS1/miR-339-5p/CDK2 axis promotes progression of ER+ breast cancer and confers tamoxifen resistance. Aging.

[118] Jiang M., Huang O., Xie Z., Wu S., Zhang X., Shen A., Liu H., Chen X., Wu J., Lou Y. (2014). A novel long non-coding RNA-ARA: Adriamycin resistance-associated. Biochem. Pharmacol..

[119] Chen Z., Pan T., Jiang D., Jin L., Geng Y., Feng X., Shen A., Zhang L. (2020). The lncRNA-GAS5/miR-221-3p/DKK2 Axis Modulates ABCB1-Mediated Adriamycin Resistance of Breast Cancer via the Wnt/β-Catenin Signaling Pathway. Mol. Ther. Nucleic Acids.

[120] Zeng Y., Wang G., Zhou C.-F., Zhang H.-B., Sun H., Zhang W., Zhou H.-H., Liu R., Zhu Y.-S. (2019). LncRNA Profile Study Reveals a Three-LncRNA Signature Associated With the Pathological Complete Response Following Neoadjuvant Chemotherapy in Breast Cancer. Front. Pharmacol..

[121] Chang L., Hu Z., Zhou Z., Zhang H. (2018). Linc00518 Contributes to Multidrug Resistance Through Regulating the MiR-199a/MRP1 Axis in Breast Cancer. Cell. Physiol. Biochem. Internat. J. Exp. Cell. Phys. Biochem. Pharmacol..

[122] Zhu Q.-N., Wang G., Guo Y., Peng Y., Zhang R., Deng J.-L., Li Z.-X., Zhu Y.-S. (2017). LncRNA H19 is a major mediator of doxorubicin chemoresistance in breast cancer cells through a cullin4A-MDR1 pathway. Oncotarget.

[123] Li Y., Wang Y., Wang H., Zhang L., Ding Y., Chen S., Yang Q., Chen C. (2017). Effects of lncRNA RP11-770J1.3 and TMEM25 expression on paclitaxel resistance in human breast cancer cells. Zhejiang Da Xue Xue Bao Yi Xue Ban.

[124] Ma T., Liang Y., Li Y., Song X., Zhang N., Li X., Chen B., Zhao W., Wang L., Yang Q. (2020). LncRNA LINP1 confers tamoxifen resistance and negatively regulated by ER signaling in breast cancer. Cell. Signal..

[125] Yao N., Fu Y., Chen L., Liu Z., He J., Zhu Y., Xia T., Wang S. (2019). Long non-coding RNA NONHSAT101069 promotes epirubicin resistance, migration, and invasion of breast cancer cells through NONHSAT101069/miR-129-5p/Twist1 axis. Oncogene.

[126] Gao H., Hao G., Sun Y., Li L., Wang Y. (2018). Long noncoding RNA H19 mediated the chemosensitivity of breast cancer cells via Wnt pathway and EMT process. OncoTarg. Ther..

[127] Yan L., Yang S., Yue C.-X., Wei X.-Y., Peng W., Dong Z.-Y., Xu H.-N., Chen S.-L., Wang W.-R., Chen C.-J. (2020). Long noncoding RNA H19 acts as a miR-340-3p sponge to promote epithelial-mesenchymal transition by regulating YWHAZ expression in paclitaxel-resistant breast cancer cells. Environ. Toxicol..

[128] Zhang X., Wang M., Sun H., Zhu T., Wang X. (2018). Downregulation of LINC00894-002 Contributes to Tamoxifen Resistance by Enhancing the TGF-β Signaling Pathway. Biochemistry..

[129] Li X., Wang S., Li Z., Long X., Guo Z., Zhang G., Zu J., Chen Y., Wen L. (2017). The lncRNA NEAT1 facilitates cell growth and invasion via the miR-211/HMGA2 axis in breast cancer. Int. J. Biol. Macromol..

[130] Zhang H.-Y., Liang F., Zhang J.-W., Wang F., Wang L., Kang X.-G. (2017). Effects of long noncoding RNA-ROR on tamoxifen resistance of breast cancer cells by regulating microRNA-205. Cancer Chemother. Pharmacol..

[131] Du C., Wang Y., Zhang Y., Zhang J., Zhang L., Li J. (2020). LncRNA DLX6-AS1 Contributes to Epithelial-Mesenchymal Transition and Cisplatin Resistance in Triple-negative Breast Cancer via Modulating Mir-199b-5p/Paxillin Axis. Cell Transplant..

[132] Chen Y.-M., Liu Y., Wei H.-Y., Lv K.-Z., Fu P. (2016). Linc-ROR induces epithelial-mesenchymal transition and contributes to drug resistance and invasion of breast cancer cells. Tumour Biol. J. Int. Soc. Oncodev. Biol. Med..

[133] Shi S.-J., Wang L.-J., Yu B., Li Y.-H., Jin Y., Bai X.-Z. (2015). LncRNA-ATB promotes trastuzumab resistance and invasion-metastasis cascade in breast cancer. Oncotarget.

[134] Zhang H., Zhang X.-Y., Kang X.-N., Jin L.-J., Wang Z.-Y. (2020). LncRNA-SNHG7 Enhances Chemotherapy Resistance and Cell Viability of Breast Cancer Cells by Regulating miR-186. Cancer Manag. Res..

[135] Li Z.-H., Yu N.-S., Deng Q., Zhang Y., Hu Y.-Y., Liu G., Huang K. (2020). LncRNA SNHG7 Mediates the Chemoresistance and Stemness of Breast Cancer by Sponging miR-34a. Front. Oncol..

[136] Tang L., Chen Y., Chen H., Jiang P., Yan L., Mo D., Tang X., Yan F. (2020). Promotes TGF-β-Induced Epithelial-Mesenchymal Transition and Enhances Chemoresistance in Triple-Negative Breast Cancer Cells via ANXA1. Front. Oncol..

[137] Liu C., Lu C., Yixi L., Hong J., Dong F., Ruan S., Hu T., Zhao X. (2023). Exosomal Linc00969 induces trastuzumab resistance in breast cancer by increasing HER-2 protein expression and mRNA stability by binding to HUR. Breast Cancer Res..

[138] Cairns J., Ingle J.N., Kalari K.R., Shepherd L.E., Kubo M., Goetz M.P., Weinshilboum R.M., Wang L. (2019). The lncRNA MIR2052HG regulates ERα levels and aromatase inhibitor resistance through LMTK3 by recruiting EGR1. Breast Cancer Res. BCR.

[139] Wang Z., Katsaros D., Biglia N., Shen Y., Loo L., Yu X., Lin H., Fu Y., Chu W.-M., Fei P. (2019). ERα upregulates the expression of long non-coding RNA LINC00472 which suppresses the phosphorylation of NF-κB in breast cancer. Breast Cancer Res. Treat..

[140] Basak P., Chatterjee S., Bhat V., Su A., Jin H., Lee-Wing V., Liu Q., Hu P., Murphy L.C., Raouf A. (2018). Long Non-Coding RNA H19 Acts as an Estrogen Receptor Modulator that is Required for Endocrine Therapy Resistance in ER+ Breast Cancer Cells. Cell. Physiol. Biochem. Internat. J. Exp. Cell. Phys. Biochem. Pharmacol..

[141] Gooding A.J., Zhang B., Gunawardane L., Beard A., Valadkhan S., Schiemann W.P. (2019). The lncRNA BORG facilitates the survival and chemoresistance of triple-negative breast cancers. Oncogene.

[142] Dong H., Wang W., Mo S., Liu Q., Chen X., Chen R., Zhang Y., Zou K., Ye M., He X. (2018). Long non-coding RNA SNHG14 induces trastuzumab resistance of breast cancer via regulating PABPC1 expression through H3K27 acetylation. J. Cell. Mol. Med..

[143] Pan Y., Pan Y., Cheng Y., Yang F., Yao Z., Wang O. (2018). Knockdown of LncRNA MAPT-AS1 inhibites proliferation and migration and sensitizes cancer cells to paclitaxel by regulating MAPT expression in ER-negative breast cancers. Cell Biosci..

[144] Peng W.-X., Huang J.-G., Yang L., Gong A.-H., Mo Y.-Y. (2017). Linc-RoR promotes MAPK/ERK signaling and confers estrogen-independent growth of breast cancer. Mol. Cancer.

[145] Xue X., Yang Y.A., Zhang A., Fong K.W., Kim J., Song B., Li S., Zhao J.C., Yu J. (2016). LncRNA HOTAIR enhances ER signaling and confers tamoxifen resistance in breast cancer. Oncogene.

[146] Zhuang Y., Nguyen H.T., Burow M.E., Zhuo Y., El-Dahr S.S., Yao X., Cao S., Flemington E.K., Nephew K.P., Fang F. (2015). Elevated expression of long intergenic non-coding RNA HOTAIR in a basal-like variant of MCF-7 breast cancer cells. Mol. Carcinog..

[147] Lin X., Dinglin X., Cao S., Zheng S., Wu C., Chen W., Li Q., Hu Q., Zheng F., Wu Z. (2020). Enhancer-Driven lncRNA BDNF-AS Induces Endocrine Resistance and Malignant Progression of Breast Cancer through the RNH1/TRIM21/mTOR Cascade. Cell Rep..

[148] Godinho M.F.E., Sieuwerts A.M., Look M.P., Meijer D., Foekens J.A., Dorssers L.C.J., van Agthoven T. (2010). Relevance of BCAR4 in tamoxifen resistance and tumour aggressiveness of human breast cancer. Br. J. Cancer.

[149] Gottesman M.M., Fojo T., Bates S.E. (2002). Multidrug resistance in cancer: Role of ATP-dependent transporters. Nat. Rev. Cancer.

[150] Dean M., Rzhetsky A., Allikmets R. (2001). The human ATP-binding cassette (ABC) transporter superfamily. Genome Res..

[151] Dean M. (2009). ABC transporters, drug resistance, and cancer stem cells. J. Mammary Gland Biol. Neoplasia.

[152] Zhang Z., Xiong R., Li C., Xu M., Guo M. (2019). LncRNA TUG1 promotes cisplatin resistance in esophageal squamous cell carcinoma cells by regulating Nrf2. Acta Biochim. Biophys. Sin..

[153] Song L., Zhou Z., Gan Y., Li P., Xu Y., Zhang Z., Luo F., Xu J., Zhou Q., Dai F. (2019). Long noncoding RNA OIP5-AS1 causes cisplatin resistance in osteosarcoma through inducing the LPAATβ/PI3K/AKT/mTOR signaling pathway by sponging the miR-340-5p. J. Cell. Biochem..

[154] Mou S.-J., Yang P.-F., Liu Y.-P., Xu N., Jiang W.-W., Yue W.-J. (2020). BCLAF1 promotes cell proliferation, invasion and drug-resistance though targeting lncRNA NEAT1 in hepatocellular carcinoma. Life Sci..

[155] Martin-Orozco E., Sanchez-Fernandez A., Ortiz-Parra I., Ayala-San Nicolas M. (2019). WNT Signaling in Tumors: The Way to Evade Drugs and Immunity. Front. Immunol..

[156] Kalluri R., Weinberg R.A. (2009). The basics of epithelial-mesenchymal transition. J. Clin. Investing..

[157] Sommers C.L., Heckford S.E., Skerker J.M., Worland P., Torri J.A., Thompson E.W., Byers S.W., Gelmann E.P. (1992). Loss of epithelial markers and acquisition of vimentin expression in adriamycin- and vinblastine-resistant human breast cancer cell lines. Cancer Res..

[158] Arumugam T., Ramachandran V., Fournier K.F., Wang H., Marquis L., Abbruzzese J.L., Gallick G.E., Logsdon C.D., McConkey D.J., Choi W. (2009). Epithelial to mesenchymal transition contributes to drug resistance in pancreatic cancer. Cancer Res..

[159] McConkey D.J., Choi W., Marquis L., Martin F., Williams M.B., Shah J., Svatek R., Das A., Adam L., Kamat A. (2009). Role of epithelial-to-mesenchymal transition (EMT) in drug sensitivity and metastasis in bladder cancer. Cancer Metastasis Rev..

[160] Huang J., Li H., Ren G. (2015). Epithelial-mesenchymal transition and drug resistance in breast cancer (Review). Int. J. Oncol..

[161] De Craene B., Berx G. (2013). Regulatory networks defining EMT during cancer initiation and progression. Nat. Rev. Cancer.

[162] Kim H.-J., Litzenburger B.C., Cui X., Delgado D.A., Grabiner B.C., Lin X., Lewis M.T., Gottardis M.M., Wong T.W., Attar R.M. (2007). Constitutively active type I insulin-like growth factor receptor causes transformation and xenograft growth of immortalized mammary epithelial cells and is accompanied by an epithelial-to-mesenchymal transition mediated by NF-kappaB and snail. Mol. Cell. Biol..

[163] Timmerman L.A., Grego-Bessa J., Raya A., Bertrán E., Pérez-Pomares J.M., Díez J., Aranda S., Palomo S., McCormick F., Izpisúa-Belmonte J.C. (2004). Notch promotes epithelial-mesenchymal transition during cardiac development and oncogenic transformation. Genes. Dev..

[164] Yook J.I., Li X.-Y., Ota I., Fearon E.R., Weiss S.J. (2005). Wnt-dependent regulation of the E-cadherin repressor snail. J. Biol. Chem..

[165] Syn W.-K., Jung Y., Omenetti A., Abdelmalek M., Guy C.D., Yang L., Wang J., Witek R.P., Fearing C.M., Pereira T.A. (2009). Hedgehog-mediated epithelial-to-mesenchymal transition and fibrogenic repair in nonalcoholic fatty liver disease. Gastroenterology.

[166] Yuan Y., Liao H., Pu Q., Ke X., Hu X., Ma Y., Luo X., Jiang Q., Gong Y., Wu M. (2020). miR-410 induces both epithelial-mesenchymal transition and radioresistance through activation of the PI3K/mTOR pathway in non-small cell lung cancer. Signal. Transduct. Target. Ther..

[167] Grotegut S., von Schweinitz D., Christofori G., Lehembre F. (2006). Hepatocyte growth factor induces cell scattering through MAPK/Egr-1-mediated upregulation of Snail. EMBO J..

[168] Sun X., Huang T., Liu Z., Sun M., Luo S. (2019). LncRNA SNHG7 contributes to tumorigenesis and progression in breast cancer by interacting with miR-34a through EMT initiation and the Notch-1 pathway. Eur. J. Pharmacol..

[169] Nassar D., Blanpain C. (2016). Cancer Stem Cells: Basic Concepts and Therapeutic Implications. Annu. Rev. Pathol..

[170] Shibue T., Weinberg R.A. (2017). EMT, CSCs, and drug resistance: The mechanistic link and clinical implications. Nat. Rev. Clin. Oncol..

[171] Mani S.A., Guo W., Liao M.-J., Eaton E.N., Ayyanan A., Zhou A.Y., Brooks M., Reinhard F., Zhang C.C., Shipitsin M. (2008). The epithelial-mesenchymal transition generates cells with properties of stem cells. Cell.

[172] Levina V., Marrangoni A.M., DeMarco R., Gorelik E., Lokshin A.E. (2008). Drug-selected human lung cancer stem cells: Cytokine network, tumorigenic and metastatic properties. PLoS ONE.

[173] Dallas N.A., Xia L., Fan F., Gray M.J., Gaur P., van Buren G., Samuel S., Kim M.P., Lim S.J., Ellis L.M. (2009). Chemoresistant colorectal cancer cells, the cancer stem cell phenotype, and increased sensitivity to insulin-like growth factor-I receptor inhibition. Cancer Res..

[174] Huang H., Sabari B.R., Garcia B.A., Allis C.D., Zhao Y. (2014). SnapShot: Histone modifications. Cell.

[175] Dawson M.A., Kouzarides T. (2012). Cancer epigenetics: From mechanism to therapy. Cell.

[176] Matei D., Fang F., Shen C., Schilder J., Arnold A., Zeng Y., Berry W.A., Huang T., Nephew K.P. (2012). Epigenetic resensitization to platinum in ovarian cancer. Cancer Res..

[177] Balch C., Nephew K.P. (2013). Epigenetic targeting therapies to overcome chemotherapy resistance. Adv. Exp. Med. Biol..

[178] Wilting R.H., Dannenberg J.-H. (2012). Epigenetic mechanisms in tumorigenesis, tumor cell heterogeneity and drug resistance. Drug Resist. Updates Rev. Commen. Antimicrobe. Anticancer Chemother..

[179] Kouzarides T. (2007). Chromatin modifications and their function. Cell.

[180] Whiteside T.L. (2008). The tumor microenvironment and its role in promoting tumor growth. Oncogene.

[181] Boelens M.C., Wu T.J., Nabet B.Y., Xu B., Qiu Y., Yoon T., Azzam D.J., Twyman-Saint Victor C., Wiemann B.Z., Ishwaran H. (2014). Exosome transfer from stromal to breast cancer cells regulates therapy resistance pathways. Cell.

[182] Kalluri R., LeBleu V.S. (2020). The biology function and biomedical applications of exosomes. Science.

[183] Pefanis E., Wang J., Rothschild G., Lim J., Kazadi D., Sun J., Federation A., Chao J., Elliott O., Liu Z.-P. (2015). RNA exosome-regulated long non-coding RNA transcription controls super-enhancer activity. Cell.

[184] Qu L., Ding J., Chen C., Wu Z.-J., Liu B., Gao Y., Chen W., Liu F., Sun W., Li X.-F. (2016). Exosome-Transmitted lncARSR Promotes Sunitinib Resistance in Renal Cancer by Acting as a Competing Endogenous RNA. Cancer Cell.

[185] Yu Z., Tang H., Chen S., Xie Y., Shi L., Xia S., Jiang M., Li J., Chen D. (2023). Exosomal LOC85009 inhibits docetaxel resistance in lung adenocarcinoma through regulating ATG5-induced autophagy. Drug Resist. Updates.

[186] Tong Y., Yang L., Yu C., Zhu W., Zhou X., Xiong Y., Wang W., Ji F., He D., Cao X. (2020). Tumor-Secreted Exosomal lncRNA POU3F3 Promotes Cisplatin Resistance in ESCC by Inducing Fibroblast Differentiation into CAFs. Mol. Ther. Oncol..

[187] Wang J., Lv B., Su Y., Wang X., Bu J., Yao L. (2019). Exosome-Mediated Transfer of lncRNA HOTTIP Promotes Cisplatin Resistance in Gastric Cancer Cells by Regulating HMGA1/miR-218 Axis. OncoTarg. Ther..

[188] Li Z., Niu H., Qin Q., Yang S., Wang Q., Yu C., Wei Z., Jin Z., Wang X., Yang A. (2019). lncRNA UCA1 Mediates Resistance to Cisplatin by Regulating the miR-143/FOSL2-Signaling Pathway in Ovarian Cancer. Mol. Ther. Nucleic Acids.

[189] Luo X., Wei J., Yang F.-L., Pang X.-X., Shi F., Wei Y.-X., Liao B.-Y., Wang J.-L. (2019). Exosomal lncRNA HNF1A-AS1 affects cisplatin resistance in cervical cancer cells through regulating microRNA-34b/TUFT1 axis. Cancer Cell Int..

[190] Xu C.G., Yang M.F., Ren Y.Q., Wu C.H., Wang L.Q. (2016). Exosomes mediated transfer of lncRNA UCA1 results in increased tamoxifen resistance in breast cancer cells. Eur. Rev. Med. Pharmacol. Sci..

[191] Ni C., Fang Q.-Q., Chen W.-Z., Jiang J.-X., Jiang Z., Ye J., Zhang T., Yang L., Meng F.-B., Xia W.-J. (2020). Breast cancer-derived exosomes transmit lncRNA SNHG16 to induce CD73+γδ1 Treg cells. Signal. Transduct. Target. Ther..

[192] Wang X., Pei X., Guo G., Qian X., Dou D., Zhang Z., Xu X., Duan X. (2020). Exosome-mediated transfer of long noncoding RNA H19 induces doxorubicin resistance in breast cancer. J. Cell. Physiol..

[193] Chen F., Chen J., Yang L., Liu J., Zhang X., Zhang Y., Tu Q., Yin D., Lin D., Wong P.-P. (2019). Extracellular vesicle-packaged HIF-1α-stabilizing lncRNA from tumour-associated macrophages regulates aerobic glycolysis of breast cancer cells. Nat. Cell. Biol..

[194] Zheng Z., Chen M., Xing P., Yan X., Xie B. (2019). Increased Expression of Exosomal AGAP2-AS1 (AGAP2 Antisense RNA 1) In Breast Cancer Cells Inhibits Trastuzumab-Induced Cell Cytotoxicity. Med. Sci. Monit..

[195] Kalluri R. (2016). The biology and function of exosomes in cancer. J. Clinic. Investing..

[196] Zhang Y., Li X., Kong X., Zhang M., Wang D., Liu Y., Lv K. (2020). Long non-coding RNA AK085865 ablation confers susceptibility to viral myocarditis by regulating macrophage polarization. J. Cell. Mol. Med..

[197] Ahmad I., Valverde A., Ahmad F., Naqvi A.R. (2020). Long Noncoding RNA in Myeloid and Lymphoid Cell Differentiation, Polarization and Function. Cells.

[198] Huang D., Chen J., Yang L., Ouyang Q., Li J., Lao L., Zhao J., Liu J., Lu Y., Xing Y. (2018). NKILA lncRNA promotes tumor immune evasion by sensitizing T cells to activation-induced cell death. Nat. Immunol..

[199] Hu Q., Ye Y., Chan L.-C., Li Y., Liang K., Lin A., Egranov S.D., Zhang Y., Xia W., Gong J. (2019). Oncogenic lncRNA downregulates cancer cell antigen presentation and intrinsic tumor suppression. Nat. Immunol..

[200] Zhang M., Wang N., Song P., Fu Y., Ren Y., Li Z., Wang J. (2020). LncRNA GATA3-AS1 facilitates tumour progression and immune escape in triple-negative breast cancer through destabilization of GATA3 but stabilization of PD-L1. Cell Prolif..

[201] Lai J., Chen B., Zhang G., Li X., Mok H., Liao N. (2020). Molecular characterization of breast cancer: A potential novel immune-related lncRNAs signature. J. Transl. Med..

[202] Ma W., Zhao F., Yu X., Guan S., Suo H., Tao Z., Qiu Y., Wu Y., Cao Y., Jin F. (2020). Immune-related lncRNAs as predictors of survival in breast cancer: A prognostic signature. J. Transl. Med..

[203] Jafari S.H., Saadatpour Z., Salmaninejad A., Momeni F., Mokhtari M., Nahand J.S., Rahmati M., Mirzaei H., Kianmehr M. (2018). Breast cancer diagnosis: Imaging techniques and biochemical markers. J. Cell. Physiol..

[204] Shi T., Gao G., Cao Y. (2016). Long Noncoding RNAs as Novel Biomarkers Have a Promising Future in Cancer Diagnostics. Dis. Markers.

[205] Qi P., Zhou X.-Y., Du X. (2016). Circulating long non-coding RNAs in cancer: Current status and future perspectives. Mol. Cancer.

[206] Bertos N.R., Park M. (2011). Breast cancer-one term, many entities?. J. Clinic. Inv..

[207] Wu B., Yuan Y., Han X., Wang Q., Shang H., Liang X., Jing H., Cheng W. (2020). Structure of LINC00511-siRNA-conjugated nanobubbles and improvement of cisplatin sensitivity on triple negative breast cancer. FASEB J. Off. Publ. Fed. Am. Soc. Exp. Biol..

[208] Arun G., Diermeier S., Akerman M., Chang K.-C., Wilkinson J.E., Hearn S., Kim Y., MacLeod A.R., Krainer A.R., Norton L. (2016). Differentiation of mammary tumors and reduction in metastasis upon Malat1 lncRNA loss. Genes Dev..

[209] Hao Q., Wang P., Dutta P., Chung S., Li Q., Wang K., Li J., Cao W., Deng W., Geng Q. (2020). Comp34 displays potent preclinical antitumor efficacy in triple-negative breast cancer via inhibition of NUDT3-AS4, a novel oncogenic long noncoding RNA. Cell Death Dis..

