# Correction: Ye et al. The Mechanisms of lncRNA-Mediated Multidrug Resistance and the Clinical Application Prospects of lncRNAs in Breast Cancer. *Cancers* 2022, *14*, 2101

**DOI:** 10.3390/cancers17132179

**Published:** 2025-06-27

**Authors:** Pingting Ye, Lei Feng, Shuo Shi, Chunyan Dong

**Affiliations:** Department of Oncology, Shanghai East Hospital, School of Medicine, Shanghai Key Laboratory of Chemical Assessment and Sustainability, School of Chemical Science and Engineering, Tongji University, Shanghai 200120, China; yepingting1996@163.com (P.Y.); 2005fenglei_101@163.com (L.F.)

In the original publication [1], there were several references cited that have been retracted. Corrections have been made as follows:

## Reference Updates:

The authors have deleted the following retracted references (previously cited as Refs. [5,51,57,58,64,131,148,159]). The following citations have been replaced with more recent or reliable sources:

- Ref. [64] → Updated to “Jiang, Y.; Qian, T.; Li, S.; Xie, Y.; Tao, M. Metformin reverses tamoxifen resistance through the lncRNA GAS5-medicated mTOR pathway in breast cancer. *Ann. Transl. Med*. **2022**, *10*, 366”.

- Ref. [137] → Updated to “Liu, C.; Lu, C.; Yixi, L.; Hong, J.; Dong, F.; Ruan, S.; Hu, T.; Zhao, X. Exosomal Linc00969 induces trastuzumab resistance in breast cancer by increasing HER-2 protein expression and mRNA stability by binding to HUR. *Breast Cancer Res.* **2023**, *25*, 124”.

- Ref. [185] → Updated to “Yu, Z.; Tang, H.; Chen, S.; Xie, Y.; Shi, L.; Xia, S.; Jiang, M.; Li, J.; Chen, D. Exosomal LOC85009 inhibits docetaxel resistance in lung adenocarcinoma through regulating ATG5-induced autophagy. *Drug Resist. Updates* **2023**, *67*, 100915”.

- Ref. [186] → Updated to “Tong, Y.; Yang, L.; Yu, C.; Zhu, W.; Zhou, X.; Xiong, Y.; Wang, W.; Ji, F.; He, D.; Cao, X. Tumor-Secreted Exosomal lncRNA POU3F3 Promotes Cisplatin Resistance in ESCC by Inducing Fibroblast Differentiation into CAFs. *Mol. Ther. Oncol.* **2020**, *18*, 1–13”.

With these corrections, the order of some references has been adjusted accordingly.

## Figure Revisions:

Figure 4, Figure 7 and Figure 8: Modified to reflect the latest data and ensure consistency with the updated references.

The updated figures are attached below:

**Figure 4:**
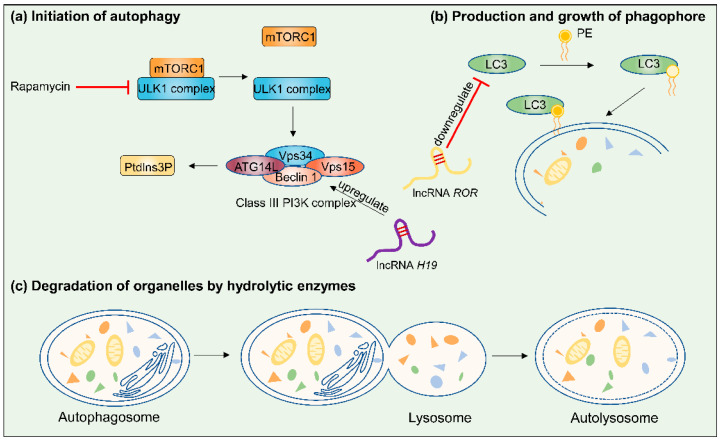
Summary of the steps involved in autophagy (lncRNA *H19* and *ROR* are used as examples for clarifying the mechanism). Autophagy is initiated by the stepwise engulfment of cellular materials by the phagophore, which sequesters materials in double-membraned vesicles known as autophagosomes [75]: (**a**) When mammalian target of rapamycin (mTOR) is inhibited, mTOR complex 1 (mTORC1) isolates from the ULK1 complex. The first step of vesicle nucleation is activating Vps34, a class III phosphatidylinositol 3-kinase (PI3K), to produce phosphatidylinositol-3-phosphate (PtdIns3P). (**b**) A part of the vesicle elongation process is to bind phosphatidylethanolamine (PE) to LC3. (**c**) The formation of autophagosomes is completed after closure of the phagophore double membrane, and then autophagosomes fuse with lysosomes, resulting in degradation of the contents.

**Figure 7:**
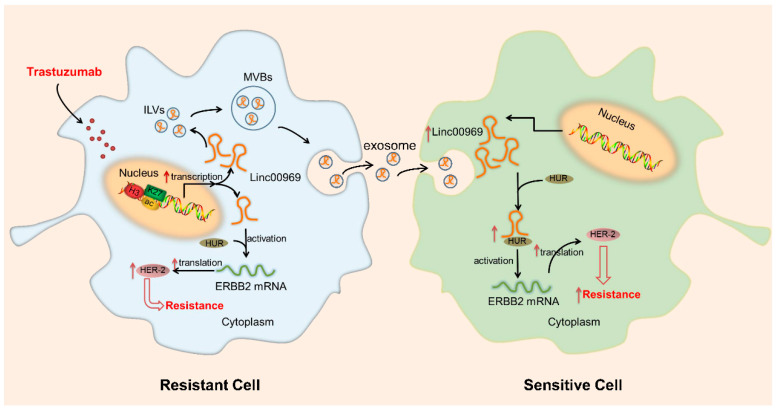
Scheme of the proposed mechanism related to *Linc00969* in trastuzumab-resistant breast cells. *Linc00969* induces trastuzumab resistance by binding to the HUR protein and promoting the translation of ERBB2 mRNA. In addition, extracellular *Linc00969* from trastuzumab-resistant cells was packaged into exosomes and disseminated trastuzumab resistance in trastuzumab-sensitive cells [137]. ILVs, intraluminal vesicles; MVBs, multivesicular bodies; HUR, Hu antigen R.

**Figure 8:**
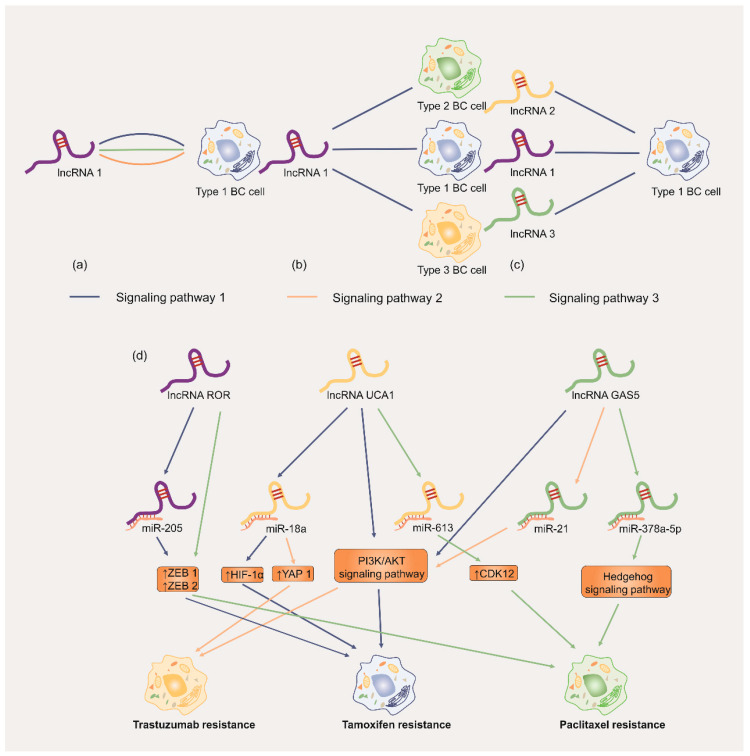
Brief sketch map of our conclusions in this review: (**a**) A certain lncRNA regulates chemoresistance in a subtype of BC cell via various signaling pathways; (**b**) a certain lncRNA induces different subtypes of BC cells to resist chemotherapeutic agents via the same signaling pathway; (**c**) a certain subtype of BC cell is regulated by various lncRNAs via the same signaling pathway; (**d**) the lncRNAs *UCA1*, *ROR*, and *GAS5* are used as examples to provide a further detailed explanation.

## Table Revisions:

Table 1, Table 2 and Table 3: Modified to reflect the latest data and ensure consistency with the updated references.

The updated tables are attached below:

**Table 1:** The role of lncRNAs in regulating cell survival and death in chemoresistant breast cancers.

Function	LncRNA	Type	Genomic Location	Expression Level *	Resistant Drugs	Cell Lines	Possible Mechanism ^§^	References
Suppressing apoptosis	*GAS5*	Tumor suppressor	chr1q25.1	↓	paclitaxel; cisplatin	MDA-MB-231; BT549	↑ miR-378a-5p/↓ SUFU signaling	[49]
	*MEG3*	Tumor suppressor	chr14q32	↓	doxorubicin; paclitaxel	Hs578T; MCF-7; MDA-MB-231	↑ TGF-β and N-cadherin protein; ↓ MMP 2, ZEB 1 and COL3A1 expression; ↓ miR-4513/↑ PBLD axis	[50,51]
	*PTENP1*	Tumor suppressor	N/A °	↓	adriamycin	MDA-MB-231; T-47D; MCF-7	↑ miR-20a/↓ PTEN axis; ↑ PI3K/AKT pathway	[52]
	*UCA1*	Oncogene	chr19q13.12	↑	tamoxifen	MCF-7; T-47D; LCC2; LCC9	↑ EZH2/↓ p21 axis; ↑ PI3K/AKT pathway; ↑ mTOR pathway	[53,54]
	*H19*	Oncogene	chr11p15.5	↑	paclitaxel	MDA-MB-453; MDA-MB-157; MDA-MB-231; ZR-75-1; MCF-7	↑ AKT pathway; ↓ BIK; ↓ NOXA	[47,48]
	*PRLB*	Oncogene	chr8p11.21	↑	5-fluorouracil	MDA-MB-231	↓ miR-4766-5p/↑ SIRT1 axis	[55]
	*LINP1*	Oncogene	chr10	↑	doxorubicin;5-fluorouracil	MDA-MB-231; MDA-MB-468; MCF-7	↓ p53; ↓ E-cadherin; ↑ N-cadherin; ↑ vimentin; ↓ caspase9/Bax	[56]
	*LOC645166*	Oncogene	N/A	↑	adriamycin	MDA-MB-231; MCF-7	↑ NF-κB/GATA3 axis	[57]
Autophagy	*EGOT*	Tumor suppressor	N/A	↓	paclitaxel	MCF-7; T-47D; UACC-812; SK-BR-3; HCC70; MDA-MB-453; MDA-MB-231; MDA-MB-468; BT549; Hs578T	↑ ITPR1	[58]
	*ROR*	Oncogene	chr18q21.31	↑	tamoxifen	BT474	↑ MDR1 and GST-π mRNA; ↓ LC3 and Beclin 1	[59]
	*H19*	Oncogene	chr11p15.5	↑	tamoxifen	MCF-7	H19/SAHH/DNMT3B axis; ↑ Beclin1	[60]
	*ZNF649-AS1*	Oncogene	chr19q13.41	↑	trastuzumab	SK-BR-3; BT474	↑ ATG5 through associating with PTBP1	[61]
	*ASAH2B-2*	Oncogene	N/A	↑	everolimus	BT474; MCF-7	↑ mTOR pathway	[62]
DNA repair	*HCP5*	Oncogene	N/A	↑	cisplatin	MDA-MB-231	↓ PTEN	[63]
	*PTENP1*	Tumor suppressor	N/A	↓	adriamycin	MDA-MB-231; T-47D; MCF-7	↑ miR-20a/↓ PTEN axis; ↑ PI3K/AKT pathway	[52]
	*GAS5*	Tumor suppressor	chr1q25.1	↓	tamoxifen	MCF-7	↑ AKT/mTOR pathway; ↓ PTEN	[64]
	*UCA1*	Oncogene	chr19q13.12	↑	trastuzumab	SKBR-3	↓ miR-18a/↑ Yes-associated protein 1 (YAP1); ↓ PTEN; ↑ CD6	[65]
	*UCA1*	Oncogene	chr19q13.12	↑	paclitaxel	MCF-7	↓ miR-613/↑ CDK12 axis	[66]
	*GAS5*	Tumor suppressor	chr1q25.1	↓	trastuzumab; lapatinib	SKBR-3	↑ miR-21; ↓ PTEN; ↑ mTOR; ↑ Ki-67	[67]
	*LINC-PINT*	Tumor suppressor	N/A	↓	paclitaxel	MDA-MB-231; BT-20	↑ NONO	[68]
	*H19*	Oncogene	chr11p15.5	↑	doxorubicin	MCF-7	↓ PARP1	[69]
	*lncMat2B*	Oncogene	N/A	↑	cisplatin	MDA-MB-231; MCF-7	N/A	[70]
	*ADAMTS9-AS2*	Tumor suppressor	N/A	↓	tamoxifen	MCF-7	↑ microRNA-130a-5p; ↓ PTEN	[71]

* The expression in resistant BC lines is indicated by arrows; ↑ for higher expression and ↓ for lower expression. **^§^** The effect of lncRNAs on associated pathways, miRNAs, genes, or transcription factors involved in resistance mechanisms are indicated by arrows: ↑ induction and ↓ repression. ° N/A, information not available.

**Table 2:** The function of lncRNAs in chemoresistant breast cancers, including regulating cell cycle, drug efflux metabolism, EMT, and epigenetic alteration.

Function	LncRNA	Type	Genomic Location	Expression Level *	Resistant Drugs	Cell Lines	Possible Mechanism ^§^	References
regulating cell cycle	*TMPO-AS1*	Oncogene	N/A °	↑	tamoxifen	MCF-7	stabilize ESR1 mRNA	[109]
	*CASC2*	Oncogene	N/A	↑	paclitaxel	MDA-MB-231; MCF-7	↓ miR-18a-5p/↑ CDK19 axis	[110]
	*LINC00511*	Oncogene	chr17q24.3	↑	paclitaxel	MDA-MB-231; MCF-7; T-47D; Hs-578T	↓ miR-29c/↑ CDK6 axis	[104]
	*NEAT1*	Oncogene	N/A	↑	cisplatin/taxol	MDA-MB-231	N/A	[111]
	*LOL*	Oncogene	N/A	↑	tamoxifen	MCF-7	↓ let-7 miRNA; ↓ ERα signaling	[112]
	*UCA1*	Oncogene	chr19q13.12	↑	tamoxifen	MCF-7; T-47D; LCC2; LCC9; BT474	↑ EZH2/↓ p21 axis; ↑ PI3K/AKT pathway; ↓ miR-18a/↑ HIF1α	[54,113]
	*DSCAM-AS1*	Oncogene	chr21q22.3	↑	tamoxifen	MCF-7; T-47D; SK-BR-3; MDA-MB-231	↑ epidermal growth factor receptor pathway substrate 8 (EPS8); ↑ ESR1; ↑ ERα; ↓ miR-137	[114,115]
	*FTH1P3*	Oncogene	N/A	↑	paclitaxel	MCF-7; MDA-MB-231; MDA-MB-468; MDA-MB-453	↓ miR-206/↑ ABCB1	[116]
	*MAFG-AS1*	Oncogene	N/A	↑	tamoxifen	MCF-7; BT474; T-47D; MCF10A	↓ miR-339-5p/↑ CDK2 axis	[117]
	*PRLB*	Oncogene	chr8p11.21	↑	5-fluorouracil	MDA-MB-231	↓ miR-4766-5p/↑ SIRT1 axis	[55]
	*UCA1*	Oncogene	chr19q13.12	↑	trastuzumab	SKBR-3	↓ miR-18a/↑ Yes-associated protein 1 (YAP1); ↓ PTEN; ↑ CD6	[65]
	*LINP1*	Oncogene	chr10	↑	doxorubicin; 5-fluorouracil	MDA-MB-231; MDA-MB-468; MCF-7	↓ p53; ↓ E-cadherin; ↑ N-cadherin; ↑ vimentin; ↓ caspase9/Bax	[56]
	*TROJAN*	Oncogene	N/A	↑	palbociclib	MCF7; T47D	↑ NKRF/CDK2 axis	[5]
	*DILA1*	Oncogene	N/A	↑	tamoxifen	MCF-7; 293-T; T47D	↑ Cyclin D1	[4]
	*ARA*	Oncogene	Xq23	↑	adriamycin	MCF-7	multiple signaling pathways	[118]
drug efflux metabolism	*GAS5*	Tumor suppressor	chr1q25.1	↓	adriamycin	MCF-7	↑ miR-221-3p/↑ Dickkopf 2 (DKK2) axis; ↑ Wnt/b-catenin pathway	[119]
	*BC032585*	Tumor suppressor	chr9	↓	taxane; anthracyclines	MDA-MB-231	↑ MDR1	[120]
	*Linc00518*	Oncogene	chr6	↑	multidrugadriamycin; vincristine; paclitaxel	MCF-7	↓ miR-199a/↑ MRP1 axis	[121]
	*FTH1P3*	Oncogene	N/A	↑	paclitaxel	MCF-7; MDA-MB-231; MDA-MB-468; MDA-MB-453	↓ miR-206/↑ ABCB1	[116]
	*ROR*	Oncogene	chr18q21.31	↑	tamoxifen	BT474	↑ MDR1 and GST-π mRNA; ↓ LC3 and Beclin 1	[59]
	*H19*	Oncogene	chr11p15.5	↑	doxorubicin; anthracyclines	MCF-7	↑ CUL4A-ABCB1/MDR1 pathway	[122]
	*RP11-770J1.3* *TMEM25*	Oncogene	N/A	↑	paclitaxel	MCF-7	↑ MRP, BCRP and MDR1/P-gp	[123]
EMT	*LINP1*	Oncogene	chr10	↑	tamoxifen	MCF-7; T-47D	↓ ER expression signaling pathway	[124]
	*MEG3*	Tumor suppressor	chr14q32	↓	doxorubicin	Hs578T	↑ TGF-β and N-cadherin protein; ↓ MMP 2, ZEB 1 and COL3A1 expression	[50]
	*NONHSAT101069*	Oncogene	chr5	↑	epirubicin	MCF-7	↓ miR-129-5p/↑ Twist1 axis	[125]
	*NEAT1*	Oncogene	N/A	↑	cisplatin/taxol	MDA-MB-231	N/A	[111]
	*H19*	Oncogene	chr11p15.5	↑	tamoxifen; paclitaxel	SK-BR-3; MCF-7	↑ Wnt pathway; ↓ miR-340-3p/YWHAZ axis	[126,127]
	*PRLB*	Oncogene	chr8p11.21	↑	5-fluorouracil	MDA-MB-231	↓ miR-4766-5p/↑ SIRT1	[55]
	*LINC00894002*	Tumor suppressor	X chromosome	↓	tamoxifen	MCF-7	↓ miR200/↑ TGFβ2 signaling pathway; ↑ ZEB1	[128]
	*LINP1*	Oncogene	chr10	↑	doxorubicin; 5-fluorouracil	MDA-MB-231; MDA-MB-468; MCF-7	↓ p53; ↓ E-cadherin; ↑ N-cadherin; ↑ vimentin; ↓ caspase9/Bax	[56]
	*NEAT1*	Oncogene	N/A	↑	5-fluorouracil	MCF-7; T-47D; MDA-MB-231; ZR-75-1	↓ miR-211/↑ HMGA2 axis	[129]
	*ROR*	Oncogene	chr18q21.31	↑	tamoxifen	MDA-MB-231; MCF-7	↓ microRNA-205; ↓ E-cadherin; ↑ vimentin; ↑ ZEB1 and ZEB2	[130]
	*DLX6-AS1*	Oncogene	N/A	↑	cisplatin	HCC1599; MDA-MB-231; HCC1806; Hs578T	↓ miR-199b-5p/paxillin signaling	[131]
	*ROR*	Oncogene	chr18q21.31	↑	5-fluorouracil; paclitaxel	T-47D; MCF-7; SK-BR-3; Bcap-37; MDA-MB-231; MCF10A	↓ E-cadherin; ↑ vimentin and N-cadherin	[132]
	*ATB*	Oncogene	chr14q11.2	↑	trastuzumab	SKBR-3	↓ miR-200c; ↑ TGF-β signaling; ↑ ZEB1 and ZNF-217	[133]
	*SNHG7*	Oncogene	chr9q34.3	↑	trastuzumab; adriamycin; paclitaxel	SKBR3; AU565; MDA-MB-231; MCF10A; MCF-7	↓ miR-186; ↓ miR-34a	[134,135]
	*DCST1-AS1*	Oncogene	N/A	↑	doxorubicin; paclitaxel	MDA-MB-231; BT-549; T-47D; MCF-7	↑ TGF-β/Smad signaling through ANXA1	[136]
epigenetic alteration	*LINC00969*	Oncogene	N/A	↑	trastuzumab	SKBR-3; BT474	↑ translation and stability of ERBB2 mRNA	[137]
	*TMPO-AS1*	Oncogene	N/A	↑	tamoxifen	MCF-7	stabilize ESR1 mRNA	[109]
	*ZNF649-AS1*	Oncogene	chr19q13.41	↑	trastuzumab	SK-BR-3; BT474	↑ ATG5 through associating with PTBP1	[61]
	*MIR2052HG*	Oncogene	N/A	↑	aromatase inhibitor	MDA-MB-231; CAMA-1; Au565; 293-T; MCF-7	↑ LMTK3; ↓ AKT/FOXO3-mediated ESR1 transcription; ↓ PKC/MEK/ERK/RSK1 pathway; ↓ ERα degradation	[138]
	*LINC00472*	Tumor suppressor	N/A	↓	tamoxifen	MCF-7; T-47D; MDA-MB-231; Hs578T	↑ phosphorylation NF-κB	[139]
	*UCA1*	Oncogene	chr19q13.12	↑	tamoxifen	MCF-7; T-47D; LCC2; LCC9	↑ EZH2/↓ p21 axis; ↑ PI3K/AKT pathway	[54]
	*H19*	Oncogene	chr11p15.5	↑	tamoxifen; fulverstrant	LCC2; LCC9; MCF-7	↑ ERα; ↑ Notch, HGF and c-MET signaling	[140]
	*BORG*	Oncogene	N/A	↑	doxorubicin	D2.OR; 67NR; 4T07; 4T1	↑ NF-κB signaling; ↑ RPA1	[141]
	*SNHG14*	Oncogene	chr15q11.2	↑	trastuzumab	SKBR-3; BT474	↑ PABPC1; ↑ Nrf2 pathway	[142]
	*MAPT-AS1*	Oncogene	chr17q21.31	↑	paclitaxel	MDA-MB-231; MDA-MB-468	↑ MAPT mRNA	[143]
	*Linc-RoR*	Oncogene	N/A	↑	tamoxifen	MCF-7	↑ MAPK/ERK signaling; ↑ ER signaling; ↓ DUSP7	[144]
	*HOTAIR*	Oncogene	chr12q13.13	↑	tamoxifen; TNF-a	MCF-7; T-47D	↑ ER signaling; ↑ SRC and p38MAPK kinases; ↑ EZH2	[145,146]
	*H19*	Oncogene	chr11p15.5	↑	paclitaxel	ZR-75-1; MCF-7	↓ BIK; ↓ NOXA	[48]
	*BDNF-AS*	Oncogene	chr11p14.1	↑	tamoxifen	MCF-7; T-47D; MDA-MB-231	↑ RNH1/TRIM21/mTOR	[147]
	*BCAR4*	Oncogene	chr16p13.13	↑	tamoxifen	ZR-75-1	↑ ERBB2/ERBB3 pathway; ↑ AKT	[148]

* The expression in resistant BC lines is indicated by arrows: ↑ for higher expression and ↓ for lower expression. **^§^** The effect of lncRNAs on associated pathways, miRNAs, genes, or transcription factors involved in resistance mechanisms are indicated by arrows: ↑ induction and ↓ repression. ° N/A, information not available.

**Table 3:** The role of exosomal lncRNAs in drug resistance in breast cancers.

LncRNA	Type	Genomic Location	Expression Level *	Resistant Drugs	Cell Lines	Possible Mechanism ^§^	Reference
*LINC00969*	Oncogene	N/A	↑	trastuzumab	SKBR-3; BT474	↑ translation and stability of ERBB2 mRNA	[137]
*H19*	Oncogene	chr11p15.5	↑	doxorubicin	MCF-7; MDA-MB-231	N/A °	[192]
*HISLA*	Oncogene	chr14q31.3	↑	docetaxel	MDA-MB-231; BT-474; MDA-MB-468; MCF-7	inhibit the hydroxylation and degradation of HIF-1α	[193]
*AGAP2-AS1*	Oncogene	chr12q14.1	↑	trastuzumab	SKBR-3; BT474	N/A	[194]
*UCA1*	Oncogene	chr19q13.12	↑	tamoxifen	MCF-7; LCC2	↓ cleaved caspase-3	[190]

* The expression in resistant BC lines is indicated by arrows: ↑ for higher expression and ↓ for lower expression. **^§^** The effect of lncRNAs on associated pathways, miRNAs, genes, or transcription factors involved in resistance mechanisms are indicated by arrows: ↑ induction and ↓ repression. ° N/A, information not available.

## Text Revisions:

The text has been modified to reflect the latest data and ensure consistency with the updated references.

The correction has been made to Section 5.1.3. Activating DNA Repair; Section 5.3. Drug Efflux; Section 5.5. Epigenetic Modification; Section 5.6. Modifying the TME via Exosomal lncRNAs; and Section 7.1. Association of lncRNAs and Patients with BC.

The correct sections are shown below:

### *5.1.3. Activating DNA Repair* 

Considerable evidence supports that many chemotherapeutic agents exert anticancer effects by destroying the stability of genes and activating downstream DNA damage signaling pathways [78,79]. Tumor cells may activate DNA damage repair pathways to resist DNA damage and contribute to MDR [80]. Accumulating studies have reported that lncRNAs in different human cancers are related to DNA repair in MDR [81–83]. It is widely accepted that phosphatase and tensin homolog (PTEN) controls DNA repair [84,85] and exerts multiple nuclear functions [86,87]. Moreover, it also participates in the key processes of genetic transmission to promote the fidelity of DNA replication [88–90] and chromosome segregation [91–93]. Jiang et al. and Li et al. reported that lncRNA growth arrest-specific transcript 5 (*GAS5*) functions as a tumor suppressor in chemoresistant BC. *GAS5* induced resistance to chemotherapeutic drugs by suppressing PTEN in two situations (different molecular subtypes of BCs and different drugs) [64,67]. In addition to PTEN, poly (ADP-ribose) polymerase (PARP) also participates in the DNA repair process, serving as an enzyme to repair single-stranded breaks [94]. Wang et al. reported that *H19* plays a crucial role in doxorubicin-resistant BC by downregulating PARP1 [69]. In the clinic, resistance to PARP inhibitors is common. Ideally, knockdown of *H19* might increase the sensitivity of BC cells to doxorubicin and PARP inhibitors. This implies that targeting lncRNAs could reverse resistance, increase the effectiveness of treatment strategies, and achieve good clinical efficacy. As shown in Table 1, although many lncRNAs participate in DNA repair via distinct pathways in the chemoresistance of BC, they are the tip of the iceberg. It is impossible to achieve clinical translation based on the currently available information. There is still a long way to go to fully clarify the relationship between lncRNAs and DNA repair.

### 5.3. Drug Efflux

Drug efflux is regarded as the predominant cause of MDR in human cancers. Hydrophobic chemotherapeutic drugs can be pumped out of tumor cells via the ATP-binding cassette (ABC) transporter superfamily, thereby reducing the effectiveness of the drugs and possibly resulting in tumor recurrence [149]. To date, according to their sequence homology and structural similarities, a total of 48 human ABC transporter genes have been divided into seven subfamilies (ABCA to ABCG) [150]. Among the ABC transporter superfamily, P-glycoprotein (P-gp/ABCB1), multidrug resistance protein 1 (MRP1/ABCC1), and breast cancer resistance protein (BCRP/ABCG2) are considered to be the most closely related to MDR in cancer cells [149,151]. Recently, a number of studies have shown that lncRNAs play a key role in increasing the outflow of a wide range of chemotherapeutic agents from human cancer cells, such as esophageal squamous cell carcinoma [152], osteosarcoma [153], and hepatocellular carcinoma [154]. A similar function of lncRNAs has also been explored in BC (Table 2). For instance, Chen et al. found that *GAS5* was downregulated in adriamycin-resistant BC cells, while the mRNA ABCB1 was upregulated based on the RNA expression profiles. Further investigating the related mechanism in detail, *GAS5* regulates its target Dickkopf 2 (DKK2) by working as a molecular sponge of miR-221-3p and inhibiting activation of the Wnt/β-catenin pathway [119]. The promoter of the ABCB1 gene contains TCF4/LEF binding motifs, which are targets of β-catenin/TCF4 transcriptional regulators [155]. Therefore, the downregulation of *GAS5* will disinhibit the Wnt/β-catenin pathway, increase the expression of ABCB1, and promote the exit of adriamycin from intracellular sources. With the function of regulating drug efflux metabolism, targeting lncRNAs may become a promising approach to eliminate or suppress MDR by reducing drug efflux from tumor cells. Ideally, the combination of chemotherapeutic drugs and lncRNA target drugs can reduce the dose and side effects for BC patients.

### 5.5. Epigenetic Modification

Epigenetic factors, such as chromatin remodeling and DNA methylation, are related to the spatial and temporal regulation of gene expression [174,175]. Therefore, a malignant phenotype may be induced by aberrant expression patterns or genomic alterations in chromatin remodelers. Although it has been reported that epigenetic factors contribute greatly to drug tolerance [176–178], most of the exact mechanisms behind these associations remain elusive. In this section, we summarized that lncRNAs regulate gene expression via epigenetic modification in chemoresistant BC cells (Table 2). As shown in Figure 7, the expression of *Linc00969* was upregulated in trastuzumab-resistant cells [137]. Then, *Linc00969* could increase the translation of ERBB2 mRNA by binding to the Hu antigen R (HUR) protein. Therefore, the protein level of HER-2 was upregulated and, subsequently, induced trastuzumab resistance in HER-2+ BC cells. As one of the most common epigenetic modifications, histone acetylation can neutralize lysine’s positive charge to relax the chromatin structure and enhance transcriptional activity [179]. It has been reported that *ZNF649-AS1*, upregulated by H3K27ac modification, confers trastuzumab resistance by binding PTBP1 and upregulating ATG5 transcription [61]. Similarly, Dong et al. used chromatin immunoprecipitation (ChIP) assays and found that lncRNA *SNHG14* can modulate H3K27 acetylation at the promoter region of the PABPC1 gene and can increase the transcription of PABPC1 [142]. Increasing the expression of PABPC1 activates the Nrf2 pathway and, then, promotes tumorigenesis and trastuzumab resistance in BC cells. Consequently, even in the same cell line exposed to the same treatment, different lncRNAs may have similar functions (e.g., guides) via different pathways. In brief, lncRNAs could play a critical biological function in regulating the expression of genes. Further research is needed to explore the deeper underlying mechanism of epigenetic modification-related lncRNAs in MDR.

### 5.6. Modifying the TME via Exosomal lncRNAs

The TME is a complex system comprising tumor cells, stromal cells (cancer-associated fibroblasts, endothelial cells, and macrophages), extracellular matrix, and soluble factors (hormones, cytokines, and enzymes) [180]. The TME not only plays an important role in the process of tumorigenesis, proliferation, and metastasis but also has a profound impact on chemotherapeutic efficacy. Exosomes, ranging in size from 20 to 150 nm, are membrane-derived vesicles originating from endosomal multivesicular bodies (MVBs) and play an essential role in TME. They can transfer useful information from host cells to recipient cells, such as lipids, proteins, microRNAs (miRNAs), messenger RNAs (mRNAs), and lncRNAs [181–183]. Thus, exosomal lncRNAs have been investigated to explore the mechanisms of MDR in different types of tumors, such as renal cancer [184], lung cancer [185], esophageal squamous cell carcinoma [186], BC (Table 3), gastric cancer [187], ovarian cancer [188], and cervical cancer [189].

Until now, few studies have addressed the link between exosomal lncRNAs and chemoresistance in BC (Table 3). As shown in Figure 7, Liu et al. reported that exosomes from trastuzumab-resistant cells packaged extracellular *Linc00969* and transferred it to trastuzumab-sensitive cells, which also resulted in upregulation of the HER-2 protein and induced resistance of recipient cells [137]. It has been reported that exosomes produced by tamoxifen-resistant LCC2 cells containing more *UCA1* are incorporated into MCF-7 cells and then significantly increase tamoxifen resistance in ERα-positive BC cells [190]. Notably, lncRNAs in exosomes derived from chemoresistant BC cells could confer resistance to sensitive cells, even in different cell lines. Additionally, the infiltration of immune cells into TME plays an indispensable role in the anti-tumor process. Ni et al. reported that the expression of CD73 on γδT cells (a predominant type of regulatory T cells) could be upregulated by lncRNA *SNHG16*, which is transmitted via BC-derived exosomes [191]. This is closely related to unfavorable pathological characteristics and a poor prognosis of BC. Based on these reports, transmission of exosomes might provide a new idea for drug therapy, which could change the susceptibility of cells to chemotherapeutic drugs or reverse the immunosuppressive microenvironment for more effective immunotherapy.

### 7.1. Association of lncRNAs and Patients with BC

Indeed, existing studies are not limited to in vitro cellular and animal experiments. Many studies have also explored the relationship between lncRNAs and patients [119,137,192]. The following two situations are common: In the first case, significant differences were found in BC tissues from patients, and further experimental verification was carried out. For instance, Chen et al. collected 26 BC tissue samples from patients and compared the expression of *GAS5* and *ABCB1* between tissues from responders and nonresponders [119]. Then, they verified that *GAS5* and *ABCB1* expression was downregulated in chemoresistant patients and cell lines, indicating a positive correlation. In the second case, significant differences were first found by in vitro cellular and animal experiments and then further validated in BC patients. For instance, lncRNA *H19* was upregulated in doxorubicin-resistant cells, as reported by Wang et al. [192]. Then, they verified this result in BC patients by statistical analysis and reported that the exosomal lncRNA *H19* may be a noninvasive biomarker for doxorubicin-resistant BC patients. Briefly, some lncRNAs that are differentially expressed in cell lines are also consistent in the BC tissues of patients who received chemotherapy. The limitations of these studies are related to the small samples of patients and the lack of statistical analysis of sensitivity and specificity for lncRNAs serving as biomarkers. To achieve clinical application, further experiments and larger-scale clinical trials are needed.

The authors state that the scientific conclusions are unaffected. The correction was approved by the Academic Editor. The original publication has also been updated.
